# The Impact of Yeast Strains and Oenological Procedures on the Chemical Composition, Antioxidant Potential, and Aromatic Profile of Blueberry Wines

**DOI:** 10.3390/foods14223930

**Published:** 2025-11-17

**Authors:** Jiaxin Zhang, Hairu Pei, Renjie Zhou, Guoqiang Zhang

**Affiliations:** 1 College of Biological and Food Engineering, Anhui Polytechnic University, Wuhu 241000, China; 13216612321@163.com (J.Z.);; 2 Wuhu Green Food Industrial Research Institute Co., Ltd., Wuhu 241000, China

**Keywords:** mixed fermentation, aroma, *Saccharomyces cerevisiae*, *Komagataella pastoris*, blueberry wine

## Abstract

To make the flavor of blueberry wine more unique, in this study, the mixed fermentation of blueberry wine was carried out to increase its original aroma components. The basic physical and chemical properties and antioxidant capacity of blueberry wine were compared when commercial *Saccharomyces cerevisiae* was used for single fermentation, *Komagataella pastoris* was used for single fermentation, commercial *Saccharomyces cerevisiae* and *Komagataella pastoris* were used for co-fermentation, and sequential fermentation was carried out for 24 h and 48 h. Moreover, the aroma components in blueberry wine were determined by electronic nose, GC-IMS, and GC-MS. The research found that there were no significant changes in the basic physico-chemical properties such as alcohol content, reducing sugar, total phenols, and flavonoids among the five fermentation methods. However, in terms of color, commercial wine yeast fermentation had higher brightness, and the a* and b* values of co-fermentation were higher. It also possessed greater antioxidant capacity when inoculated in sequence for 24 h and 48 h. All five fruit wines were most sensitive to the W1W, W2W, W2S, W1S, and W5S sensors. GC-IMS detection found that 48 h sequential inoculation could produce new aroma components, enhancing the flavor of the fruit wine. Meanwhile, non-targeted metabolomics research by GC-MS showed that *Komagataella pastoris* could make the aroma components of blueberry fruit wine more diverse and taste more layered. This work contributes to the advancement of fruit wine flavor profile enhancement.

## 1. Introduction

The quality of wine is determined not only by factors such as fruit varieties and fermentation temperature [1], but also by the internal conditions during fermentation—primarily the secondary metabolites produced. Among these, volatile organic compounds (VOCs) exert a decisive influence on shaping the sensory characteristics of the final product. Moreover, this serves as a key determinant influencing consumer purchasing behavior [2].

Blueberry fruits, as raw materials for blueberry wine, directly affect the VOCs of blueberry wine. Blueberry fruits can develop different aromas due to their variety, their own physical and chemical properties, etc., which, in turn, affect the final blueberry wine produced through fermentation. The blueberry cultivar significantly influences the fruit’s aromatic profile. For instance, as demonstrated by Forney et al. [3], wild lowbush blueberries are characterized by esters like ethyl 2-methylbutanoate and methyl 2-methylbutanoate, which impart dominant “fruity” and “sweet” notes. In contrast, cultivated highbush varieties are dominated by monoterpenoids such as geraniol and linalool, contributing primarily “floral” and “citrus” aromas. This fundamental difference in volatile composition means that the choice of cultivar directly shapes the resulting flavor and sensory experience. The phenolic profile and bioactivity of blueberry wine are direct consequences of both the raw material and the winemaking strategy [4].

The winemaking process, particularly the choice of yeast strain and fermentation conditions, acts upon this aromatic foundation. Yeast metabolism during fermentation is critical for generating a wide array of volatile esters and higher alcohols, fundamentally shaping the final aroma. There are currently many studies focusing on strains to investigate the changes in the aroma of blueberry wine after fermentation. Liao et al. [5] found that the optimized co-culture of *L. fermentum* and *L. plantarum* not only enhanced phenolic content, but also promoted the synthesis of key aroma compounds. This specific bacterial combination led to a marked increase in fusel alcohols like 3-methyl-1-butanol and carbonyl compounds such as 2-heptanone, thereby enriching the overall aromatic complexity of the final product compared to single-strain fermentations or unfermented juice. Huang et al. [6] demonstrated that mixed fermentation of *H. uvarum* and *S. cerevisiae* effectively enriched the aroma complexity of fruit wines. This was particularly evident in blueberry wine, which achieved the highest diversity and concentration of volatile compounds among all samples, establishing it as the most notable product for aroma enhancement through this innovative method.

To maximize production efficiency, most enterprises tend to adopt standardized traditional fermentation techniques and extensively use commercial fermentation yeast on industrial-scale fruit wine production. This practice often results in a lack of flavor diversity, leading to products that are monotonous, lacking in distinctiveness, and limited in innovation. To better align with consumers’ growing demand for unique and high-quality flavor experiences, it is essential to optimize and modernize traditional fermentation methods to improve the sensory and qualitative attributes of blueberry fruit wine. Blueberries, often referred to as the “king of berries”, are highly valued for their antioxidant properties, anti-aging effects, and benefits to eyesight [7,8,9].

Once blueberries have rotted, they are often processed into other by-products to extend their market value [10,11]. They are commonly processed into various by-products such as blueberry powder, blueberry juice, and freeze-dried blueberries. Among these, blueberry fruit wine is widely loved by younger consumers in light of its mild alcohol content and sweet flavor profile.

However, blueberries naturally exhibit characteristics such as high acidity, low sugar content, and insufficient aromatic complexity, which can result in a less-than-optimal flavor profile in the final product. Furthermore, the distinctiveness and richness of the wine’s flavor are often diminished by the extensive usage of commercial yeast strains. Currently, alternative fermentation strategies—such as non-*Saccharomyces cerevisiae* fermentation, single-strain fermentation, co-fermentation, and sequential inoculation—are increasingly being explored and applied in winemaking [12,13,14]. These approaches have also been studied in other fruit wines, including those made from raspberry, pineapple, and lychee [15,16,17].

There are several analytical methods available for the detection of aroma components. By employing headspace–high-performance liquid extraction (HS-HPLE) coupled with gas chromatography–mass spectrometry (GC-MS), Qiu et al. [18] identified *Hanseniaspora occidentalis* as the non-*Saccharomyces cerevisiae* yeast strain with the most significant potential for producing fruity aromas and a higher number of esters during olive fermentation among the three strains tested. Ding et al. [19] conducted co-fermentation experiments using various species of *Komagataella pastoris* and *Saccharomyces cerevisiae*. Gas chromatography–ion mobility spectrometry (GC-IMS) was used to identify 61 volatile aroma components in total. By applying fingerprint profiling to monitor dynamic changes in key flavor compounds, the fermentation process was effectively controlled, thereby enhancing the wine’s overall quality. Yuan et al. [20] developed a hybrid from two southern highbush blueberry varieties (*Vaccinium virgatum*, *Vaccinium corymbosum*, and *Vaccinium darrowii*) using gas chromatography–high-resolution mass spectrometry (GC-QTOF-MS), refining the two analytical approaches involving both SPE and SPME with multivariate statistical analysis. Their study focused on hybrid cultivars “Misty” and “O’Neal,” highlighting the potential of integrating volatile extraction techniques with metabolic profiling tools to directly analyze compositional differences in food raw materials after undergoing complex processing steps.

In this research, the VOC profiles, flavor characteristics, and classification of blueberry wines produced using various fermentation techniques were systematically analyzed using electronic nose technology, GC–MS, HS-GC-IMS, and sensory analysis. Clarifying the effects of various processes of fermentation on the volatile makeup of blueberry wine was the main goal of this study. The findings are expected to establish a scientific foundation for strengthening the aromatic diversity and sensory characteristics of fruit wines.

Therefore, this study focuses on blueberry fruit wine and investigates four different fermentation methods: mono-fermentation, co-fermentation, and mixed fermentation with sequential inoculation at 24 h and 48 h intervals using combinations of *Saccharomyces cerevisiae* and non-*Saccharomyces cerevisiae* strains. The objective is to evaluate the impact of these fermentation methods on the physicochemical properties, bioactive compound content, antioxidant capacity, and aroma composition of blueberry fruit wine, thereby providing a scientific foundation for improving its flavor and overall quality.

## 2. Materials and Methods

### 2.1. Blueberry and Yeast Strains

The raw material, blueberries of the “Emerald” cultivar, were provided by Anhui Ziyue Biotechnology Co., Ltd. (Wuhu, China), and the soluble solids of blueberry juice were 10.5 °Brix, pH 3.26, and titratable acid 5.63 g/L. Fermentations were conducted using the commercial yeast *Saccharomyces cerevisiae* (Lamothe Abiet, Canéjan, France) and the non-*Saccharomyces* yeast *Komagataella pastoris* (CICC 32846).

### 2.2. Laboratory Scale Fermentation of Blueberry Wine

One kilogram of frozen blueberries (−20 °C storage) was thawed overnight at 4 °C under refrigerated conditions. After thawing, sound berries were manually sorted to exclude decayed fruits, pedicels, and foliar debris. The sorted berries were manually macerated to release the juice, which was then transferred into pre-sterilized 1.5 L glass fermentation vessels (one vessel per replicate). The soluble solids concentration (SSC, °Brix) of the blueberry juice was determined using a digital refractometer. Food-grade sucrose was aseptically added to adjust the SSC to 20°Brix, with gentle stirring until complete dissolution. Subsequently, 125 mg of potassium metabisulfite (to control microbial spoilage) and 30 mg of pectinase (to enhance juice yield and clarity) were accurately weighed, added to the juice, and homogenized by gentle swirling.

Activated yeast cultures were inoculated under four experimental regimens:

(1) Single-strain fermentation with commercial *Saccharomyces cerevisiae*: Inoculated at a final concentration of 5 × 10^6^ colony-forming units (cfu)·mL^−1^ using a commercial *Saccharomyces cerevisiae* strain.
(2) Single-strain fermentation with non-*Saccharomyces*: Inoculated at 5 × 10^6^ cfu·mL^−1^ using a non-*Saccharomyces* yeast strain.
(3) Sequential mixed fermentation: Initially inoculated with the non-*Saccharomyces* strain at 2.5 × 10^6^ cfu·mL^−1^, followed by inoculation with the commercial *Saccharomyces* cerevisiae at 2.5 × 10^6^ cfu·mL^−1^ after 24 h.
(4) Co-inoculated mixed fermentation: Simultaneously inoculated with both the non-*Saccharomyces* strain and commercial *Saccharomyces cerevisiae*, each at 2.5 × 10^6^ cfu·mL^−1^.

Post-inoculation, each vessel was gently agitated to ensure uniform yeast distribution and then incubated at 20 °C in a constant-temperature, constant-humidity incubator. All treatments were performed in triplicate to ensure experimental reproducibility. During fermentation, SSC (°Brix) was monitored daily using the refractometer until no significant change was observed over two consecutive measurements, indicating fermentation completion. The resulting blueberry wine was filtered through sterile cheesecloth to remove pomace and residual solids, and then decanted into sterile 50 mL centrifuge tubes and stored at −20 °C pending further analysis.

### 2.3. Determination of Physiochemical Compositions of Blueberry Wines

Following the procedure outlined in the International Organization for Vine and Wine’s 2020 edition of the International Compendium of Analytical Methods for Wine and Grape Juice, the ethanol concentration and total acid content of blueberry wine was ascertained.

The soluble solids content (SSC) was measured using a handheld refractometer (PAL-1, ATAGO, Tokyo, Japan), while the wine samples’ pH was evaluated using a pH meter (SX-610, Shanghai Sanshin Instrumentation Co., Ltd., Shanghai, China).

The reducing sugar content (RSC) in blueberry wine was quantified using the DNS method and expressed as glucose equivalents (y = 1.0308x − 0.0446, R^2^ = 0.9968). The method was adapted from Dimitra Dimitrellou [21] with minor modifications: 1.2 mL of diluted wine sample was used, followed by the sequential addition of 0.8 mL of distilled water and 1 mL of DNS reagent. The blend was thoroughly mixed, heated in a boiling water bath for 5 min, and then allowed to stand at room temperature before incorporating 1 mL of distilled water. After mixing again, the absorbance was measured at 540 nm and substituted into the standard curve for quantification.

Determination of total phenols (TPC) was performed using the Folin–Ciocalteu method, calibrated against in terms of gallic acid equivalents (mg GAE/L, y = 2.38x + 0.0458, R^2^ = 0.9958) [22].

Flavonoid content was assessed using the sodium nitrite–aluminum nitrate method described by Nardini et al. [23], with rutin as the reference standard. The corresponding calibration curve was y = 0.7108x − 0.0338 (R^2^ = 0.9982).

The content of anthocyanins in blueberry wine was determined by the pH differential method, and the experiment was carried out according to the method introduced in Ning et al. [7].

The colorimetric properties of the fermented blueberry beverage were analyzed by means of a portable colorimeter (COLOR READER CR–10 Plus, KONICA MINOLTA, INC., Tokyo, Japan), with the white calibration plate serving as the reference standard. For each replicate, 5 mL of sample was analyzed to obtain L* (lightness), a* (red/green coordinate), and b* (yellow/blue coordinate) values [24].

### 2.4. Determination of DPPH and ABTS in Blueberry Wine

The DPPH radical scavenging activity was assessed based on a modified Herald’s approach [25].

The ABTS radical scavenging capacity was assessed using a modified protocol adapted from Re’s method [26].

### 2.5. Volatile Compounds of Blueberry Wine

#### 2.5.1. Electronic Nose

The experiment employed an electronic nose system (AIRSENSE-PEN3, Schweirn, Germany) equipped with ten distinct metal–oxide–semiconductor (MOS) thin-film sensors (Table 1) to detect specific volatile compounds. The experimental procedure was as follows. Wine samples weighing exactly 10.00 g were placed in 40 mL containers. After equilibration at room temperature, the sample was injected and analyzed. Each sampling cycle lasted one second and the injection flow was 400 mL/min. The total detection time was set to 100 s, followed by a cleaning period of 120 s.

#### 2.5.2. GC-IMS

The volatile compounds in wine samples with different treatments were examined using GC-IMS (flavorspec^®^, G.A.S, Dortmund, Germany) with some modifications based on Yang et al.’s experiments [19]. The process was as follows: 2 mL of the wine sample was placed in a 20 mL headspace bottle and heated for 20 min at 60 °C while stirring at 500 rpm. The variations in drift flow and carrier flow are shown in Table 2. The ionization mode was positive. The identification of volatile compounds was achieved by leveraging the instrument’s native database (NIST library and IMS database) and plugins (Reporter, Gallery Plot, Dynamic PCA).

#### 2.5.3. GC-MS

GC-MS analysis was performed on a SHIMADZU GC-2020 system fitted with a DB-5MS capillary column. Helium, the carrier gas, flowed at 1 mL/min with a 3 mL/min inlet purge. Following a 1 μL split injection (5:1), the oven temperature was held at 50 °C for 1 min, ramped at 8 °C/min to 310 °C, and held for 11.5 min. The injection port, transfer line, and ion source were maintained at 280 °C, 280 °C, and 230 °C, respectively. Mass spectra were acquired in EI mode (−70 eV) from *m*/*z* 33-500 at 12.5 spectra/s after a 6.5 min solvent delay [27].

### 2.6. Statistical Analysis

The mean ± standard error was used to express all results, which were gathered from triplicate experiments. IBM SPSS 25.0 software was used for data analysis, and software Origin 2021 was used for plotting.

## 3. Results and Discussion

### 3.1. Physicochemical Properties of Blueberry Wines

In this study, the physicochemical properties of blueberry wines produced via five fermentation methods were dynamically monitored over 14 days. The SSC of Pk-48Sc was substantially less than that of other blueberry wines, while Sc exhibited the highest SSC (Table 3). This could be attributed to the varying fermentation capabilities of different strains. Compared to the other four blueberry wines, Sc’s pH was noticeably higher. Sc’s RSC was more than that of the other fruit wines, indicating that *Saccharomyces cerevisiae* can accelerate the fermentation rate of blueberry fruit wine. The flavonoid content of Pk-48Sc was the highest. Nevertheless, Sc-48Pk had the highest flavonoid content. The fermentation circumstances, such as yeast strains, fermentation temperature, and fermentation duration, are linked to the different phenolic components [28]. Adding *Komagataella pastoris* to blueberry fruit wines at different times results in different fermentation stages, and the produced phenolic substances and their contents will also be different, leading to the results shown in the experiment.

Regarding the color parameters of blueberry fermented wines, L*, a*, and b* values were checked following the fermentation process (*p* < 0.05) (Table 3). The L* values of the wines were arranged in ascending order as follows: Pk-48Sc < Pk-24Sc < Pk < Sc < Pk-Sc (Table 3). Additionally, the pure *Saccharomyces cerevisiae*-fermented beverage demonstrated the highest red and yellow color tones. This was followed by Pk-Sc, Pk-24Sc, Pk-48Sc, and the pure Pk-fermented wine. These findings suggest that inoculation with *Komagataella pastoris* can facilitate the shift of the beverage color towards red and yellow hues while decreasing its brightness. This phenomenon may be ascribed to differences in phenolic compounds. It is well established that anthocyanins are vital in influencing the coloration of blueberry wines. Moreover, non-anthocyanin phenolic compounds in wines, such as flavonols, 3-flavaols, and phenolic acids, can form co-pigment complexes with anthocyanins. This interaction can enhance the color stability of wines during the aging process. Phenolic compounds, such as antioxidants, can protect anthocyanins from oxygen [29,30]. In addition, the higher the anthocyanin content in blueberry wine, the darker the color of the simulated color blocks will be. Moreover, as co-pigments, phenolic compounds can interact with anthocyanins to form anthocyanin cofactor complexes, resisting the non-oxidative degradation of anthocyanins, such as hydration and further molecular splitting [31].

### 3.2. Antioxidant Property Analysis

**Figure 1:**
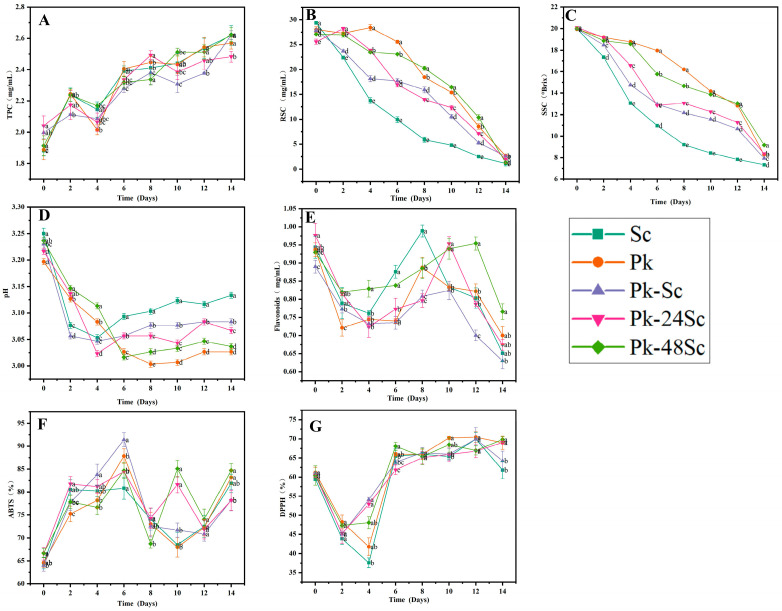
The basic physical and chemical properties and antioxidant activity of different blueberry fruit wines. TPC (**A**), RSC (**B**), SSC (**C**), pH (**D**), Flavonoids (**E**), ABTS (**F**) and DPPH (**G**).

The antioxidant activity of blueberry wines, assessed via DPPH and ABTS assays, exhibited dynamic changes throughout the 14-day fermentation, closely linked to the transformation of phenolic compounds. Although the differences among the blueberry wines were minimal, transient peaks in scavenging rates were observed on days 4 and 10 (Figure 1F,G), suggesting critical phases where yeast metabolism synergistically promoted the release or biosynthesis of phenolic antioxidants. The Pk-48Sc wine demonstrated the highest final antioxidant activity, consistent with its enriched phenolic profile. This aligns with established findings that antioxidant potential is governed by phenolic composition, particularly flavonoids and anthocyanins. Notably, fluctuations in total phenolic content (TPC), flavonoids, and phenolic acids (Figure 1A,E) correlated temporally with the antioxidant peaks, indicating that microbial activity during fermentation actively modulates phenolic bioavailability and efficacy rather than merely preserving native compounds. These results underscore the fact that fermentation strategy—specifically sequential inoculation—significantly influences the phenolic profile and its resultant antioxidant activity. The enhanced antioxidant efficacy in Pk-48Sc wine reflects a qualitative improvement in phenolic composition, achieved through yeast-driven biotransformation during fermentation [28,32,33].

### 3.3. Electronic Nose Analysis

**Figure 2:**
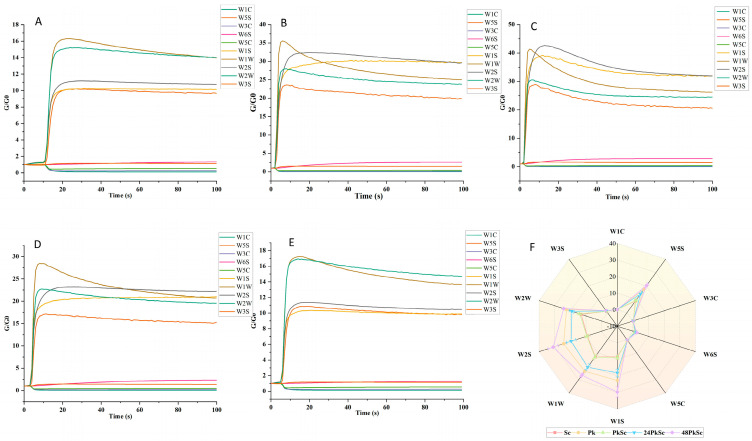
Reaction time for different sensors for different fermentation ways of blueberry wine (**A**–**E**). Radar chart of electronic nose response signal for different blueberry wine fermentation methods (**F**).

The image presents the typical response curves of the electronic nose corresponding to different treated wine samples (Figure 2). Time (in seconds) is plotted on the abscissa, and relative resistivity (G/G0) is plotted on the ordinate. The sensor conductivity in pure air is indicated by G0, whereas the sensor conductivity in the alcohol sample gas is represented by G. The response curves across the five figures exhibit similar temporal trends. Specifically, the sensors W1W, W2W, W2S, W1S, and W5S show a slight decrease after reaching their peak at 3 s and subsequently stabilize. These sensors maintained relatively high response values throughout the detection process, with G/G0 values consistently higher than those of the other five sensors. This suggests that the concentration of volatile compounds detected by these sensors was comparatively higher in the wine samples. In contrast, the response values of sensors W6C and W3S slightly increased and then plateaued, while those of W3C, W1, and W5C exhibited a slight decreasing trend.

### 3.4. GC-IMS Analysis

**Figure 3:**
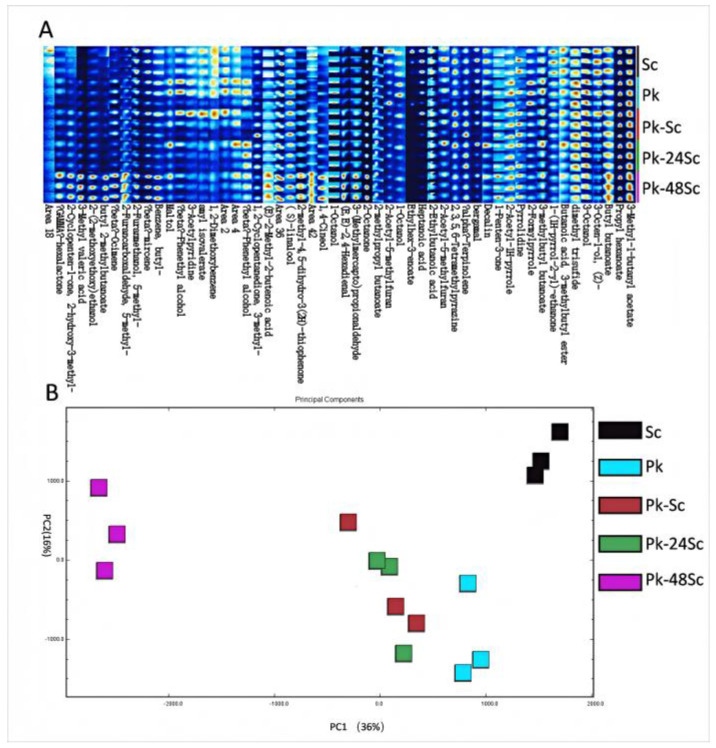
Fingerprints of the changes in volatile organic compounds during fermentation of blueberry wines as determined by GC-IMS (**A**). PCA plots of VOCs analyzed by GC-IMS of blueberry wines fermented in different ways (**B**).

As shown in Table S1, GC-IMS detected a total of 14 acid compounds, 10 alcohols, 6 ketones, 12 esters and 5 aldehydes. Among them, the increase in esters and other compounds adds a bouquet aroma to blueberry fruit wine. In addition, ester components formed in fermented foods have been reported to be unique to microbial species [34]. In the fingerprint chromatogram Figure 3A, the redder and brighter the color, the higher the concentration of these compounds, while a lower concentration corresponds to a reduced color intensity.

Principal component analysis (PCA) relies on the signal intensity of volatile compounds and can effectively differentiate wine samples (Figure 3B). The PCA results are presented in Figure 3, where black, blue, red, green, and purple squares (each representing three replicates) correspond to the key volatile components of Sc, Pk, Pk-Sc, Pk-24Sc, and Pk-48Sc, respectively. The noticeable distance between samples along the PC1 axis indicates a significant difference among them.

### 3.5. GC-MS Analysis

#### 3.5.1. Screening of Differential Metabolites

**Figure 4:**
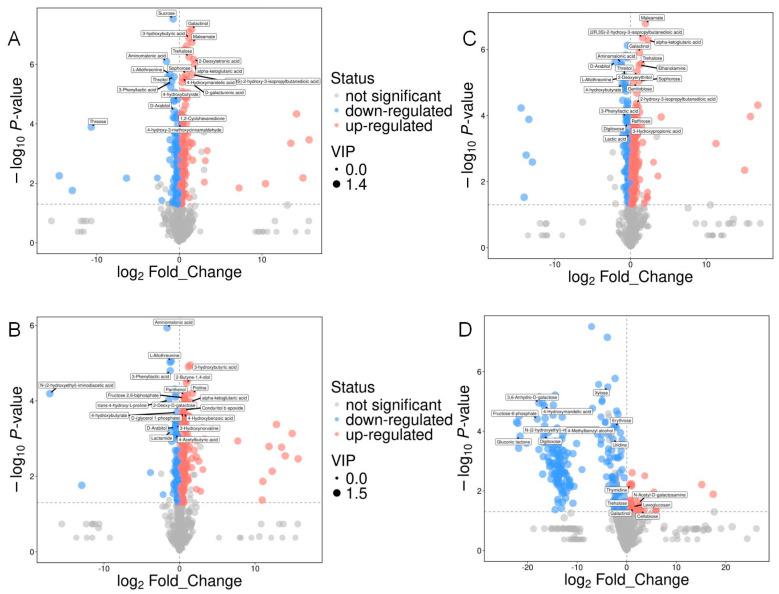
Volcano maps of blueberry wines. Sc vs. Pk (**A**), Sc vs. Pk-Sc (**B**), Sc vs. Pk-24Sc (**C**) and Sc vs. Pk-48Sc (**D**).

In this study, 240 metabolites were left after relative standard deviation de-noising. Supervised orthogonal projections to latent structures–discriminant analysis (OPLS-DA) was used to detect markedly changed metabolites and display group separation. Additionally, VIP scores from the first principal component of the OPLS-DA model were extracted to assess the contribution of each variable to the classification. Metabolites with VIP > 1 and *p*-values < 0.05 (Student’s *t*-test) were defined as significantly altered (Table S2). Based on these criteria, a total of 175 significantly down-regulated and 171 up-regulated metabolites were screened between the Pk and Sc groups. From the Pk-Sc group and the Sc group, 122 significantly down-regulated metabolites and 180 up-regulated metabolites were filtered. From the Pk-24Sc group and the Sc group, 88 significantly down-regulated metabolites and 261 up-regulated metabolites were selected. From the Pk-48Sc group and the Sc group, 188 significantly down-regulated metabolites and 31 up-regulated metabolites were screened (Figure 4).

#### 3.5.2. Analysis of VOC Changes in Blueberry Wines

**Figure 5:**
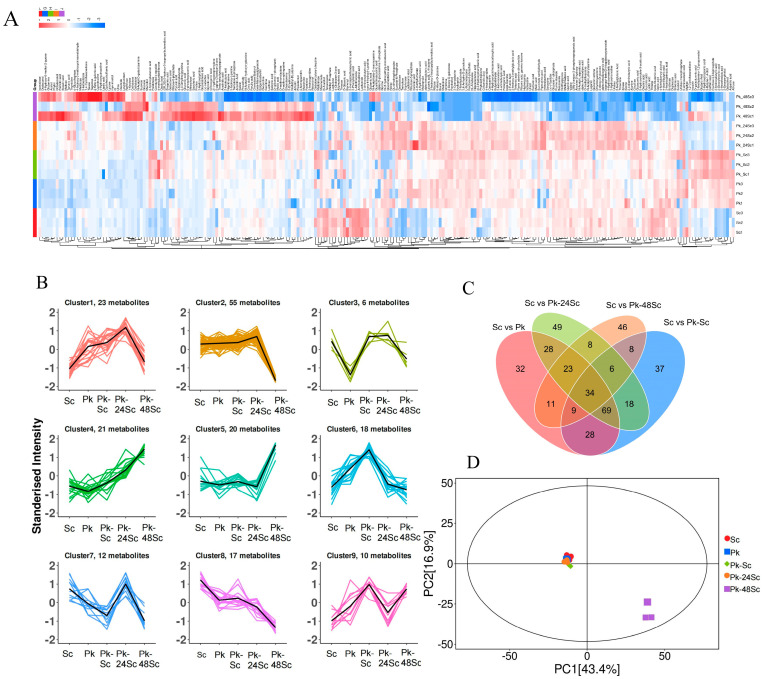
Analysis of VOC changes in blueberry wines using GC–MS. Heatmap (**A**), K-means (**B**), Venn diagram (**C**), and PCA (**D**).

Fermented blueberry fruit wine contains a large amount of acids (Figure 5A), which may be related to the fact that aldehydes can be oxidized to acids or converted to alcohols when subjected to microbial activity, which makes them unstable [35]. This is also one of the reasons for the small content and variety of aldehydes. Moreover, blueberry fruit wine contains a lot of amino acids (Table S2). During fermentation, transaminase can transform valine and isoleucine, which are branched-chain amino acids, into α-keto acids. Decarboxylation then takes place in the corresponding aromatic active aldehydes, alcohols, and carboxylic acids, resulting in a drop in concentrations [36]. Consistent with our findings, a related investigation found that decreases in valine and isoleucine led to the synthesis of molecules with odor contributions, 2-methylpropionic acid, and 2-methylbutyric acid [37].

In Figure 5C, each circle signifies a comparison group. The numerical values within the intersecting regions represent the quantity of differential metabolites common to the respective groups, whereas the values in the non-intersecting areas correspond to metabolites unique to a single group. According to prior studies, the development of food scent is intimately linked to strain species and their metabolic variations in the food matrix. Single bacteria and mixed cultures have distinct functions in altering the matrix, which could enhance the food’s aroma profile [38].

The cumulative contribution of PC1 and PC2 is 60.3% (Figure 5D). Among them, the contribution rate of PC1 is 43.4% and that of PC2 is 16.9%. The five groups of wine samples exhibit distinct distributions in the diagram. The clear separation of group Pk-48Sc from other treatment groups by the principal components implicates the 48 h sequential inoculation of *Saccharomyces cerevisiae* and non-*Saccharomyces* yeasts as a significant factor modifying the aroma compound composition in blueberry wine. Group Pk-48Sc is located at a relatively large distance from the other treatment groups, suggesting that there are notable differences in aroma compounds among them.

The relative contents of metabolites with the same change trend among the five groups were compared in order to examine the relative content change trends of metabolites in various groups. As shown in Figure 5B, the relative contents of 20 and 21 metabolites in cluster 4 and cluster 5 were relatively high in Pk-48Sc. The relative contents of metabolites in cluster 2, cluster 7, and cluster 8 were relatively low. In Pk-24Sc, the relative contents of 96 metabolites were relatively high in cluster 1, cluster 2, cluster 3, and cluster 7.

**Figure 6:**
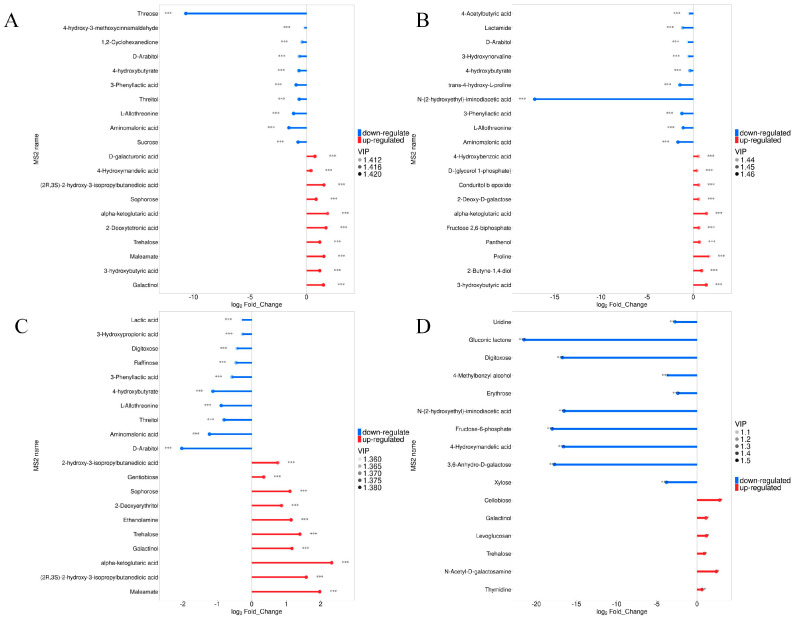
Matchstick analysis of blueberry wines. Sc vs. Pk (**A**), Sc vs. Pk-Sc (**B**), Sc vs. Pk-24Sc (**C**) and Sc vs. Pk-48Sc (**D**) (Note: * 0.01 < *p* < 0.05, ** 0.001 < *p* < 0.01, *** *p* < 0.001).

In Figure 6, the analysis shows that the differential metabolites with significant variances can either significantly activate or strongly inhibit the expression of matching enzyme genes, and the expression of some metabolites related to the topic can be verified accordingly. Compared with Sc, the relative content of threose in Pk was larger (Figure 6A), and the relative content of N-(2-hydroxyethyl)-iminodiacetic acid was the largest in Pk-Sc (Figure 6B). The relative contents of 4-hydroxybutyrate, L-Allothreonine, Threitol, Aminomalonic acid, D-Arabitol, (2R,3S)-2-hydroxy-3-isopropylbutanedioic acid, and alpha-ketoglutaric acid were highest in Pk-24Sc (Figure 6C). Pk-48Sc was the most abundant in N-(2-hydroxyethyl)-iminodiacetic acid, Fructose-6-phosphate,4-Hydroxymandeli acid,3,6-Anhydro-D-galactose, Gluconic lactone, and Digitoxose (Figure 6D).

## 4. Conclusions

In order to give blueberry fruit wine a unique aromatic composition, this study used separate fermentation and mixed fermentation with *Saccharomyces cerevisiae* and *Komagataella pastoris* to regulate the fermentation process and the production of aromatic compounds in fruit wine. Through the composition and interaction of microorganisms and the efficiency of fermentation, the physical, chemical, and sensory properties of fruit wine are changed. There were no obvious differences in physical and chemical properties such as alcohol content, RSC, and SSC. However, compared with Sc and Pk, mixed fermentation resulted in a higher content of polyphenols and flavonoids. At the same time, the DPPH and ABTS of mixed fermentation wines were also higher than those obtained by single-colony fermentation. A total of 42 compounds were detected by GC-IMS, including 14 acid compounds, 10 alcohols, 6 ketones, 12 esters and 5 aldehydes. GC-MS analysis showed that 176 compounds with VIP > 1 and *p*-values < 0.05 were screened out compared with Sc. The findings indicated that the combination of *Komagataella pastoris* and *Saccharomyces cerevisiae* in mixed fermentation positively influenced the aromatic compounds present in blueberry fruit wine, a finding that is of certain significance for further improving the flavor of fruit wine. However, it is necessary to further optimize the proportion of strains and the influence of other factors to develop a blueberry fruit wine that meets the needs of consumers.

## Figures and Tables

**Table 1:** Sensor performance description.

NO.	Sensor Name	General Description/Compounds Type
S1	W1C	Aromatic, benzene
S2	W5S	High sensitivity, sensitive to nitrogen oxides, broad range
S3	W3C	Aromatic, ammonia
S4	W6S	hydrogen
S5	W5C	Short-chain alkane aromatic components, arom–aliph
S6	W1S	broad-methane
S7	W1W	sulfur–organic
S8	W2S	Alcohols, aldehydes, ketones, broad-alcohol
S9	W2W	Aromatic ingredient, organic sulfides, sulph–chlor
S10	W3S	Long-chain alkanes, methane–aliph

**Table 2:** The variations of drift flow and carrier flow.

Time (min)	Record	E1-Drift Flow (mL/min)	E2-Carrier Flow (mL/min)
0	1	75	2
2	-	75	2
10	-	75	10
20	-	75	100
30	0	75	100

**Table 3:** The basic physical and chemical properties, antioxidant activity, and color of different blueberry fruit wines.

Parameters	Sc	Pk	Pk-Sc	Pk-24Sc	Pk-48Sc
SSC (°Brix)	5.83 ± 0.06 c	6.47 ± 0.06 a	6.37 ± 0.06 a	6.07 ± 0.06 b	6.17 ± 0.06 b
pH	3.10 ± 0.01 b	3.12 ± 0.01 a	3.09 ± 0.01 c	3.10 ± 0.01 b	3.13 ± 0.01 a
Ethanol (%)	8.08 ± 0.03 b	7.84 ± 0.03 d	8 ± 0.06 c	8.16 ± 0.03 a	8.04 ± 0.03 bc
Total acid content (g/L)	6.75 ± 0.19 d	7.69 ± 0.68 c	6.79 ± 0.25 d	8.94 ± 0.66 b	10.81 ± 0.47 a
RSC (mg/mL)	0.73 ± 0.03 ab	0.71 ± 0.02 ab	0.69 ± 0.01 b	0.71 ± 0.02 ab	0.75 ± 0.03 a
TPC (mg/mL)	2.63 ± 0.07 b	2.59 ± 0.03 b	2.58 ± 0.06 b	2.62 ± 0.04 b	2.83 ± 0.04 a
Flavonoids (mg/mL)	0.87 ± 0.01 d	1.01 ± 0.05 b	1.13 ± 0.01 a	0.94 ± 0.03 c	1.01 ± 0.01 b
DPPH (%)	61.69 ± 0.28 ab	63.47 ± 1.60 a	60.47 ± 1.14 b	63.10 ± 1.29 a	62.54 ± 0.75 ab
ABTS (%)	70.00 ± 1.321 b	72.48 ± 1.35 a	68.48 ± 0.61 b	72.48 ± 1.63 a	72.15 ± 0.41 a
Anthocyanins (g/L)	0.22 ± 0.00 a	0.12 ± 0.01 b	0.07 ± 0.00 d	0.06 ± 0.00 d	0.09 ± 0.00 c
L*	26.33 ± 0.12 a	25.87 ± 0.12 b	26.27 ± 0.06 a	25.80 ± 0.17 b	25.73 ± 0.12 b
a*	0.43 ± 0.06 a	−4.37 ± 0.06 e	−0.63 ± 0.06 b	−3.07 ± 0.12 d	−4.17 ± 0.12 c
b*	5.30 ± 0.00 a	3.97 ± 0.06 d	5.10 ± 0.17 b	4.20 ± 0.00 d	3.90 ± 0.00 c
Simulated color	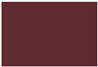	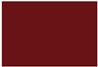	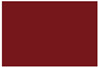	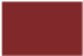	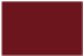

L* stands for brightness, a* for red-green degree, and b* for yellow-blue degree. Note: The signiffcant difference was *p* < 0.05.

## Data Availability

The original contributions presented in this study are included in the article/Supplementary Materials. Further inquiries can be directed to the corresponding author.

## Supplementary Materials

The following supporting information is available at https://www.mdpi.com/article/10.3390/foods14223930/s1: Table S1: Volatile organic compounds in blueberry wines detected using GC-IMS; Table S2: Significantly altered metabolites (VIP > 1, *p* < 0.05) in blueberry wines detected via GC-MS; Table S3: Relative content of compounds in blueberry wine detected by GC-MS.
