# Supplementary material for: The Impact of Yeast Strains and Oenological Procedures on the Chemical Composition, Antioxidant Potential, and Aromatic Profile of Blueberry Wines
Source: Foods. 2025 Nov 17;14(22):3930. doi: 10.3390/foods14223930 (PMC12651299; doi:10.3390/foods14223930)
Supplement: Supplementary file 1 [file foods-14-03930-s001.zip › foods-3977511-supplementary.pdf]

Table S1. Volatile organic compounds of blueberry wines via GC-IMS.

ID	Compounds	Odour	CAS	RI	Formula	MW	Rt[sec]	Dt
	Acids							
1	Heptanoic acid	Fat	111148	1057	C7H14O2	130.2	588.097	1.78913
2	3-Methyl valeric acid	Grass	105431	951.2	C6H12O2	116.2	235.909	1.60686
3	(E)-2-Methyl-2-butenoic acid	-	80591	941.8	C5H8O2	100.1	218.459	1.44564
4	2-Ethylbutanoic acid	Cheese, sour	88095	932.3	C6H12O2	116.2	202.254	1.55557
	Alcohols							
5	beta-Phenethyl alcohol	Rose	60128	1106.4	C8H10O	122.2	917.212	1.50572
6	alpha-Terpinolene	Citrus, lemon	586629	1090.7	C10H16	136.2	795.852	1.30386
7	2-(2-methoxyethoxy)ethanol	Floral	111773	988.6	C5H12O3	120.1	319.876	1.49056
8	3-Octanol	Citrus, Mushroom, Nut, Oil	589980	999.5	C8H18O	130.2	350.493	1.4004
9	1,4-Cineol	Menthol	470677	1018.7	C10H18O	154.3	416.445	1.31333
10	(S)-linalool	Coriander, Floral, Lavender, Lemon, Rose	78706	1087.9	C10H18O	154.3	776.504	1.21311
11	(Z)-3-Octen-1-ol	Dust, Toasted Nut	20125842	1057	C8H16O	128.2	588.097	1.36027
12	5-methyl-2-Furanmethanol	Sweet,caramel	3857258	964.4	C6H8O2	112.1	262.709	1.55099

13	1-Octanol-D	Fat,citrus	111875	1074.7	C8H18O	130.2	735.288	1.47274
14	1-Octanol-M	Fat,citrus	111875	1057	C8H18O	130.2	689.255	1.46643
	Ketones							
15	2-Octanone	Apple,woody,herbal	111137	1000	C8H16O	128.2	352.107	1.74106
16	3-methyl-1,2-Cyclopentanedione	sweet,caramel,maple sugar,coffee,,woody	765708	1090.9	C6H8O2	112.1	797.61	1.51128
17	1-(1H-pyrrol-2-yl)-ethanone	-	1072839	1069.3	C6H7NO	109.1	656.862	1.49166
18	1-Penten-3-one	Pungent,peppery mustard,garlic onion	1629589	691.1	C5H8O	84.1	35.791	1.32039
19	2-methyl-4,5-dihydro-3(2H)-thiophenone	Cabbage, Must, Onion	13679851	1000	C5H8OS	116.2	352.129	1.53388
20	2-hydroxy-3-methyl-2-Cyclopenten-1-one	caramellic	80717	1030.3	C6H8O2	112.1	462.261	1.51635
	Esters							
21	amyl isovalerate	Apple,fresh fruity	25415627	1114.8	C10H20O2	172.3	989.325	1.48165
22	2-methylbutanoate	Fruity	15706737	1038.2	C9H18O2	158.2	496.6	1.39811
23	3-methylbutyl butanoate	Banana,fruity	106274	1051.7	C9H18O2	158.2	557.408	1.40442
24	GAMMA-hexalactone	-	695067	1050.7	C6H10O2	114.1	555.703	1.52845
25	acetic acid hexyl ester	Fruity	626777	1084.9	C9H18O2	158.2	755.398	1.83723
26	3-Methyl-1-butanyl acetate	Banana	123922	871.6	C7H14O2	130.2	125.677	1.32595

27	dimethyl trisufide	Alliaceous,onion	3658808	969.3	C2H6S3	126.3	273.304	1.30274
28	2-methylpropyl butanoate	Sweet,fruity	539902	956.6	C8H16O2	144.2	246.504	1.34763
29	Ethylhex-3-enoate	Fruit	2396830	1019.1	C8H14O2	142.2	417.907	1.76121
30	acetic acid hexyl ester	Fruit	142927	1020	C8H16O2	144.2	421.319	1.41716
31	Butyl butanoate	Floral	109217	984.6	C8H16O2	144.2	309.593	1.33249
32	3-methylbutyl ester Butanoic acid	Banana	109193	1011.8	C9H18O2	158.238	391.379	1.37929
	Aldehydes							
33	3-(Methylmercapto)propionaldehyde	Cooked potato, Soy	3268493	916.3	C4H8OS	104.2	177.562	1.39268
34	5-methyl-2-Furancarboxaldehyde	caramellic	620020	951.5	C6H6O2	110.1	236.533	1.46671
35	(E,E)-2,4-Hexadienal	Green	142836	918.7	C6H8O	96.1	181.064	1.44931
36	bergamal	Fresh,lemon	106729	1051.2	C9H16O	140.2	557.977	1.70293
37	2-Formylpyrrole	Musty,Beefy,coffee	1003298	1044.7	C5H5NO	95.1	526.152	1.47484
	Others							
38	3-Acetylpyridine	Savory	350038	1115.2	C7H7NO	121.1	992.843	1.58721
39	2-Acetyl-1H-pyrrole	roast	1072839	1088.6	C6H7NO	109.1	781.321	1.47379
40	2,3,5,6-Tetramethylpyrazine	Cocoa, Coffee, Green, Mocha, Roast	1124114	1091.4	C8H12N2	136.2	801.128	1.69833
41	Maltol	caramellic	118718	1126.3	C6H6O3	126.1	1096.615	1.53906

42	Decalin	Floral	91178	1062.5	C10H18	138.3	617.649	1.2478
43	butyl-Benzene	-	104518	1056.7	C10H14	134.2	586.392	1.56419
44	1,2-Dimethoxybenzene	-	91167	1149.8	C8H10O2	138.2	1355.167	1.58906
45	Pyrrolidine	Ammoniacal,egg amine	123751	697	C4H9N	71.1	37.253	1.25088
46	beta-Ocimene	Citrus,Floral	13877913	1039.1	C10H16	136.2	500.578	1.27197
47	beta-mircene	spicy	123353	988.3	C10H16	136.2	319.175	1.63692
48	2-Acetyl-5-methylfuran-D	Nut, Vegetable	1193799	1050.5	C7H8O2	124.1	554.567	1.58521
49	2-Acetyl-5-methylfuran-M	Nut, Vegetable	1193799	1019.5	C7H8O2	124.1	419.369	1.56284

By using n-alkanes (C4-C9) as an external reference, the relative retention index (RI)values of VOCs in wine samples were determined. Odor descriptions were cited from http://www.chemicalbook.com,http://www.odour.org.uk,http://www.flavornet.org and https://www.femaflavor.org/flavor-library.

Table S2. The metabolites with VIP>1 and p<0.05 (student t test) of blueberry wines via GC-MS.

ID	Aroma compound	rt	CAS	VIP (Pk vs Sc)	P-value (Pk vs Sc)	VIP (Pk-Sc vs Sc)	P-value (Pk-Sc vs Sc)	VIP (Pk-24Sc vs Sc)	P-value (Pk-24Sc vs Sc)	VIP (Pk-48Sc vs Sc)	P-value (Pk-48Sc vs Sc)
	Acids										
1	3-Aminoisobutyric acid	10.96	144-90-1	1.13	0.045457638	1.42	0.001187576	1.21	0.014306619	1.53	0.002746722
2	(2R)-2-amino-3-phosphonopropanoic acid	19.25	20263-06-3	1.15	0.049905081			1.17	0.036964845	1.53	0.001090187
3	Glucosaminic acid	23.21	3646-68-2	1.16	0.044271762	1.38	0.003613398				
4	Caffeic acid	24.51	331-39-5	1.19	0.033321645	1.29	0.019708845	1.33	0.002978867		
5	4-Acetylbutyric acid	13.09	3128-6-1	1.19	0.03040024	1.43	0.000751044	1.32	0.003521687		
6	Glycolic acid	8.53	79-14-1	1.20	0.033444252	1.30	0.013856696				
7	Fumaric acid	13.50	110-17-8	1.26	0.018464114	1.40	0.002695556	1.32	0.004304531		
8	Pelargonic acid	13.69	112-05-0	1.27	0.019847011	1.41	0.001391527				
9	Sinapinic acid	25.71		1.22	0.025600321	1.46	0.001695135				
10	Saccharic acid	22.93	576-42-1	1.22	0.025600321	1.43	0.000880614				
11	2-Keto-L-gulonic acid	21.46	342385-52-8	1.22	0.025600321	1.43	0.000880614				
12	Palmitic acid	23.47	1957-10-3	1.27	0.016849217						
13	Galactonic acid	22.82	576-36-3	1.27	0.022947191						
14	Arachidic acid	27.84	506-30-9	1.31	0.008135926						
15	Glutamic acid	17.79	6893-26-1	1.32	0.008980157	1.31	0.012621813	1.30	0.006595352		
16	Quinic acid	21.04	77-95-2	1.33	0.007421303			1.28	0.009186297	1.33	0.019839737
17	Phosphate	12.10	7664-38-2	1.33	0.005603875	1.27	0.025093734	1.35	0.00086417		
18	beta-Glutamic acid	17.58	1948-48-7	1.34	0.001344987			1.34	0.001344987		
19	Pipecolinic acid	13.72	535-75-1	1.35	0.006314702	1.32	0.018799515	1.35	0.000383771		
20	6-phosphogluconic acid	27.45	53411-70-4	1.36	0.008298027						

21	Stearic acid	25.74	1957-11-4	1.37	0.00269204						
22	N-(2-hydroxyethyl)-iminodiacetic acid	20.62	93-62-9	1.37	0.00245793	1.46	6.45088E-05	1.31	0.004913105	1.53	6.45091E-05
23	3-Hydroxypropionic acid	9.80	503-66-2	1.38	0.000856234	1.44	0.000370808	1.37	0.00028643		
24	Lactic acid	8.22	50-21-5	1.39	0.00063397			1.31	0.005504925		
25	Glutaric Acid	14.42	110-94-1	1.40	0.000201744	1.43	0.001135631	1.20	0.024669475		
26	Citramalic acid	15.45	2306-22-1	1.40	0.000381579			1.37	0.000160918		
27	Gluconic acid	22.81	526-95-4	1.40	0.000430467	1.44	0.000302726				
28	2-Furoic Acid	9.65	88-14-2	1.41	9.56761E-05	1.39	0.003544762	1.36	0.000386021		
29	4-Hydroxybenzoic acid	17.90	99-96-7	1.41	9.41564E-05	1.44	0.000208502	1.37	0.000381589		
30	Mucic acid	23.30	526-99-8	1.41	4.84177E-05	1.36	0.006826867	1.31	0.005263515		
31	(2R,3S)-2-hydroxy-3-isopropylbutanedioic acid	16.96	921-28-8	1.42	2.2074E-06	1.46	0.003937545	1.38	4.41208E-07		
32	alpha-ketoglutaric acid	17.10	328-50-7	1.42	9.5505E-07	1.45	0.000105939	1.38	5.21066E-07		
33	3-Phenyllactic acid	17.21	828-01-3	1.42	3.78609E-06	1.46	1.59694E-05	1.37	0.000108143		
34	Chlorogenic Acid	33.88	327-97-9	1.42	1.28076E-05	1.45	0.006286672				
35	D-galacturonic acid	22.35	685-73-4	1.42	3.10167E-06	1.45	0.006765692				
36	4-Hydroxymandelic acid	19.80	1198-84-1	1.42	2.8122E-06			1.38	0.001766201	1.53	1.39735E-05
37	2-Deoxytetronic acid	14.82	51267-44-8	1.42	6.75491E-07	1.46	0.002059291	1.37	0.000439731		
38	3-hydroxybutyric acid	10.09	306-31-0	1.42	1.33894E-07	1.46	1.12866E-05	1.37	0.000110292		
39	Aminomalonic acid	15.42	1068-84-4	1.42	7.94316E-07	1.46	1.16455E-06	1.38	2.53806E-06		
40	3-Hexenedioic acid	15.99	4436-74-2	1.42	0.000454542	1.46	0.003063132	1.37	0.008219789		
41	Maleamate	16.72	557-24-4	1.42	1.81315E-07	1.44	0.012626857	1.38	1.63233E-07		
42	Glutaconic acid	19.4	1724-02-3			1.20	0.036918166	1.24	0.016695942	1.12	0.035929017
43	Threonic acid	16.81	7306-96-9			1.26	0.031103928	1.32	0.003630257		
44	Salicylic acid	16.08	69-72-7			1.27	0.026859913	1.14	0.03909686		
45	2,4-diaminobutyric acid	15.21	305-62-4			1.28	0.022635476				
46	Succinic acid	12.91	110-15-6			1.29	0.021844991				
47	Iminodiacetic acid	16.39	142-73-4			1.32	0.014305462	1.38	5.42628E-05		
48	Pyruvic acid	8.04	127-17-3			1.33	0.014758228	1.36	0.000340296		
49	2-mercaptoethanesulfonic acid	17.63	3375-50-6			1.33	0.022709828			1.11	0.019059714

50	5-Hydroxyindole-3-acetic acid	25.41	54-16-0			1.36	0.008600348				
51	Oxamic acid	11.67	471-47-6			1.38	0.00482122				
52	Oxalic acid	9.52,0	144-62-7			1.38	0.006035574				
53	Itaconic acid	13.34	97-65-4			1.43	0.000258104				
54	2-methylfumarate	14.34	498-24-8			1.46	0.0004225				
55	Lactobionic Acid	30.75	96-82-2					1.01	0.044555077		
56	Lyxonic acid, 1,4-lactone	19.41	15384-34-6					1.18	0.028770581		
57	Gallic acid	22.32	149-91-7					1.20	0.025557212	1.50	0.000282732
58	Ferulic acid	24.00	1135-24-6					1.20	0.023818955		
59	alpha-Aminoadipic acid	18.91	542-32-5					1.27	0.010557368	1.53	0.002635169
60	Lipoic acid	22.39	1077-28-7					1.28	0.007153654		
61	4-Hydroxycyclohexanecarboxylic acid	15.79	3685-22-1					1.31	0.003231421		
62	2-Methylglutaric Acid	14.54	617-62-9					1.36	0.000488719	1.53	0.000395028
63	Malic acid	15.73	97-67-6					1.37	8.22778E-05		
64	2-hydroxy-3-isopropylbutanedioic acid	17.06	16048-89-8					1.38	4.34645E-05		
65	alpha-ketoisocaproic acid	10.47	816-66-0							1.45	0.000689822
66	3-hydroxybenzoic acid	16.98	1999-6-9							1.53	0.011061656
67	N-Acetyl-L-aspartic acid	18.38	997-55-7							1.53	0.002335989
68	Shikimic acid	20.17	138-59-0							1.53	0.007004716
	Carbohydrate										
1	Maltotriose	38.75	1109-28-0	1.14	0.033300396			1.18	0.019665818	1.36	0.009906416
2	Lactose	30.30	63-42-3	1.21	0.026502585						
3	Levoglucosan	18.81	498-07-7	1.21	0.033327384			1.30	0.006433336	1.48	0.03409434
4	Glucose	21.68	50-99-7	1.22	0.025600321	1.43	0.000880614				
5	Trehalose-6-phosphate	34.73	4484-88-2	1.24	0.03442317	1.24	0.03442317				
6	Xylose	18.24	6763-34-4	1.24	0.021065949	1.36	0.006130135	1.36	0.00047805	1.48	3.27853E-06
7	N-Acetyl-D-galactosamine	23.76	14215-68-0	1.25	0.026794037	1.38	0.00652386	1.37	0.008642005	1.52	0.025032867
8	d-Glucoheptose	24.44	62475-58-5	1.31	0.010987152	1.41	0.00247838	1.36	0.010896253		
9	2-Deoxy-D-galactose	20.18	1949-89-9	1.32	0.008560719	1.44	0.000159601	1.29	0.008559608	1.53	0.000735964
10	Lyxose	18.22	1114-34-7	1.33	0.005132774						

11	Fructose 2,6-biphosphate	25.30	79082-92-1	1.34	0.005812742			1.29	0.004827033		
12	Fructose-6-phosphate	26.36	643-13-0	1.35	0.004170336	1.41	0.002087556	1.33	0.002789798	1.53	3.88827E-05
13	Gentiobiose	31.34	554-91-6	1.36	0.003097567	1.32	0.016456569	1.38	1.03737E-05		
14	Cellobiose	30.24	528-50-7	1.36	0.002899058					1.51	0.045321956
15	Raffinose	36.40	512-69-6	1.37	0.001844356	1.38	0.003374334	1.37	0.000136249		
16	Ribose	18.33	24259-59-4	1.39	0.000845563			1.34	0.002089852	1.30	0.015572806
17	Isomaltose	32.07	499-40-1	1.39	0.000784341			1.19	0.02775202		
18	Melibiose	31.78	66009-10-7	1.40	0.000330894			1.21	0.020486403		
19	3,6-Anhydro-D-galactose	19.60	14122-18-0	1.40	0.000313647					1.53	1.12221E-05
20	Palatinose	31.40	15132-06-6	1.41	5.04323E-05	1.42	0.001322356	1.30	0.003936528		
21	6-deoxy-D-glucose	19.10	488-79-9	1.41	9.48244E-05			1.34	0.001497159		
22	Maltose	30.53	69-79-4	1.41	6.27702E-05	1.35	0.047273746	1.37	0.000201723		
23	Trehalose	30.65	99-20-7	1.42	5.57159E-07	1.45	0.0002406	1.38	2.71768E-06	1.48	0.030272239
24	Sophorose	30.94	534-46-3	1.42	2.08149E-06	1.44	0.010059413	1.38	9.06844E-06		
25	Sucrose	29.64	57-50-1	1.42	2.96852E-08	1.41	0.014006395	1.31	0.02089113		
26	Ribulose-5-phosphate	23.98	4151-19-3	1.42	0.000342606	1.40	0.002723004				
27	Threose	15.24	95-44-3	1.42	0.000128642						
28	N-Methyl-DL-alanine	10.34	600-21-5			1.20	0.049009159				
29	alpha-D-glucosamine 1-phosphate	20.26	2152-75-2			1.26	0.026452512	1.21	0.023512546		
30	Glucose-1-phosphate	19.84	59-56-3			1.35	0.009284381				
31	Melezitose	37.69	597-12-6			1.37	0.004010905	1.29	0.008627993		
32	Sucrose-6-Phosphate	32.98	36064-19-4					1.02	0.031594973		
33	Lactulose	30.18	4618-18-2					1.33	0.030150744		
34	1-Kestose	36.54	470-69-9					1.34	0.001785788		
35	Tagatose	20.89	87-81-0					1.34	0.001134517		
36	Digitoxose	17.55	527-52-6					1.37	0.00016713	1.53	0.000139888
37	Erythrose	15.15	583-50-6							1.51	6.78252E-05
38	2-deoxy-D-glucose	20.01	154-17-6							1.53	0.024505529
	amino acid										
1	O-methylthreonine	10.38	2076-57-5	1.13	0.047604241						

2	Valine	11.09	72-18-4	1.25	0.013142701	1.33	0.015298677			1.18	0.003248174
3	N-Acetyl-L-phenylalanine	20.15	2018-61-3	1.28	0.015472689	1.23	0.040015723				
4	Glutamine	16.53	56-85-9	1.31	0.007611358			1.34	0.001832172		
5	Ornithine	20.43	70-26-8	1.32	0.009746772	1.42	0.001571078			1.53	0.000657282
6	Canavanine	11.27	543-38-4	1.33	0.004976131	1.36	0.005536158	1.33	0.002181911		
7	Alanine	9.02	56-41-7	1.34	0.002435408						
8	Threonine	14.16	72-19-5	1.35	0.004132807			1.36	0.009875486	1.06	0.001081376
9	Oxoproline	16.26	98-79-3	1.37	0.00105184						
10	trans-4-hydroxy-L-proline	16.31	51-35-4	1.40	0.000344109	1.45	9.63817E-05	1.29	0.00779476	1.53	0.001409654
11	L-Allothreonine	14.17	28954-12-3	1.42	2.09157E-06	1.46	8.86226E-06	1.38	6.94174E-06		
12	Proline	12.60	147-85-3			1.44	6.36933E-05				
13	Cycloleucine	10.84	52-52-8					1.21	0.022563106		
14	Isoleucine	12.50	73-32-5					1.27	0.010849956		
15	3-hydroxy-L-proline	15.31						1.25	0.018826047		
16	3-Cyanoalanine	13.96	6232-19-5							1.53	0.001286729
	Alcohol										
1	D-erythro-sphingosine	27.76	123-78-4	1.24	0.032169348			1.15	0.043781863		
2	Acetol	18.99	116-09-6	1.28	0.013406271			1.30	0.005909676		
3	Phytol	24.87	150-86-7	1.26	0.024387796						
4	Conduritol b epoxide	22.21	6090-95-5	1.34	0.00623677	1.45	0.000174988	1.33	0.002957644		
5	myo-inositol	23.91	87-89-8	1.35	0.002680864						
6	2-Butyne-1,4-diol	11.46	110-65-6	1.36	0.002341106	1.45	3.34213E-05	1.33	0.002103472		
7	Cholestan-3beta-ol	34.90	80-97-7	1.36	0.007918166	1.36	0.007918166				
8	3-Methylamino-1,2-propanediol	10.93	40137-22-2	1.39	0.000679956	1.42	0.001429628				
9	Panthenol	21.24	16485-10-2	1.41	0.000138177			1.35	0.001173547	1.53	0.000369615
10	Mannitol	21.93	87-78-5	1.41	0.000118216	1.39	0.003425985				
11	D-Arabitol	19.15	488-82-4	1.41	3.7018E-05	1.43	0.000514334	1.38	2.3721E-06		
12	2-Deoxyerythritol	12.43	3068-00-6	1.42	9.00146E-06	1.46	0.001635453	1.38	4.62455E-06		
13	Threitol	15.88	7493-90-5	1.42	2.62052E-06	1.40	0.002183028	1.38	6.25049E-06	1.37	0.011703224
14	4-Methylbenzyl alcohol	11.86	589-18-4			1.38	0.005505302	1.23	0.018636211	1.01	0.000132426

15	5-Dihydrocortisol	34.48	516-41-6					1.14	0.031276843		
16	Dodecanol	16.84	112-53-8					1.22	0.024178499		
17	DL-dihydrosphingosine	28.37	13552-09-5					1.38	0.00011029		
	Ketone										
1	4-methylumbelliferone	23.24	90-33-5	1.22	0.025600321						
2	L-Gulonolactone	21.69	1128-23-0	1.24	0.024297705					1.53	0.001166509
3	Gluconic lactone	21.58	90-80-2	1.26	0.019094889			1.29	0.008286518	1.53	0.000145112
4	D-erythronolactone	14.76	15667-21-7	1.26	0.018147914	1.34	0.015075893	1.34	0.00166167		
5	4-Hydroxy-6-methyl-2-pyrone	15.82	675-10-5	1.28	0.013673169			1.33	0.002370116		
6	4-Cholesten-3-one	35.01	601-57-0	1.31	0.013044482					1.11	0.016755303
7	D-(glycerol 1-phosphate)	19.74	1957-3-4	1.32	0.00829977	1.44	0.000199851				
8	1,2-Cyclohexanedione	18.48	765-87-7	1.41	6.07227E-05	1.29	0.022014894	1.33	0.000802195		
9	21-hydroxypregnenolone	33.52	1164-98-3	1.43	0.000880614	1.43	0.000880614				
10	Dihydroxyacetone	11.75	62147-49-3			1.40	0.018174862	1.33	0.020767021		
	aldehyde										
1	4-hydroxy-3-methoxycinnamaldehyde	22.44	458-36-6	1.41	0.000101783	1.25	0.029381816	1.34	0.00125278		
2	Succinate semialdehyde	10.63	692-29-5			1.35	0.006778708	1.18	0.031240402		
	others										
1	Inosine	29.02	58-63-9	1.28	0.018819304						
2	Uridine monophosphate	31.52	58-97-9	1.42	0.000447592						
3	Loganin	32.22	18524-94-2	1.39	0.000882754	1.27	0.022911266			1.44	0.001937498
4	Galactinol	32.25	3687-64-7	1.42	6.52391E-08	1.46	0.003344809		1.22388E-06	1.48	0.035716279
5	Lactamide	10.15	2043-43-8	1.40	0.000724641	1.44	0.000631952	1.36	0.000483233	1.20	0.000531117
6	Ethanolamine	12.01	141-43-5	1.40	0.000471077	1.27	0.025953065	1.38	2.93336E-06		
7	4-hydroxybutyrate	11.48	502-85-2	1.42	1.5784E-05	1.45	0.000204337	1.38	1.10408E-05		
8	4-hydroxypyridine	10.23	626-64-2	1.41	8.23448E-05	1.44	0.000286364	1.36	0.000672383		
9	4-Hydroxyquinazoline	16.68	491-36-1	1.27	0.015908378	1.30	0.017965867	1.35	0.001105215		
10	Atrazine-2-hydroxy	21.85	2163-68-0	1.26	0.020589805	1.33	0.013429826	1.28	0.007438133	1.53	0.000408852
11	(+/-)-Taxifolin	32.52	480-18-2	1.37	0.006658618	1.37	0.006658618				
12	Oxamide	13.07	471-46-5	1.31	0.008081282			1.21	0.024669828		

13	Isopropyl-beta-D-thiogalactopyranoside	23.66	367-93-1	1.20	0.036254869			1.30	0.005021967	1.44	0.012220955
14	Thymidine	17.64	50-89-5			1.25	0.037948118	1.36	0.000640605	1.47	0.00595707
15	Salicin	28.30	138-52-3					1.29	0.008443908		
16	Purine riboside	26.99						1.35	0.000750566		
17	Uridine	28.00	58-96-8					1.31	0.00650188	1.37	0.000184184
18	Maleimide	8.90	541-59-3					1.28	0.009766799		
19	N-Acetyl-beta-D-mannosamine	24.10	7772-94-3					1.28	0.008183397		
20	N-Acetyl-5-hydroxytryptamine	28.46	1210-83-9					1.20	0.026035073		
21	Naphthalene	11.14	91-20-3					1.11	0.040480929		
22	Malonamide	14.89	108-13-4							1.53	0.006800199
23	3-Methyloxindole	18.97	1504-06-9							1.53	0.000943957
24	Tetracosane	27.32	646-31-1							1.50	0.000257478
25	Kyotorphin	28.97	70904-56-2							1.53	0.00041991

Table S3. Relative content of compounds in blueberry wine detected by GC-MS.

ID	Compounds	Rt	Mass	CAS	Sc	Pk	Pk-Sc	Pk-24Sc	Pk-48Sc
	**Acids**								
1	2-Furoic Acid	9.65294,0	125	88-14-2	0.000069±0.000002a	0.000091±0.000001a	0.000096±0.000007a	0.000087±0.000002b	0.000036±0.000031b
2	2-hydroxybutanoic acid	9.45,0	131	565-70-8	0.000006±0.000006c	0.00005±0.000001a	0.000062±0.000002a	0±0b	0.000033±0.000015b
3	2-ketoadipate	11.6439,0	71	3184-35-8	0.000149±0.000029a	0.000136±0.000008a	0.000149±0.000036a	0.000187±0.000018a	0.000208±0.000116a
4	2-ketobutyric acid	8.93647,0	89	600-18-0	0.000057±0.000009a	0.000058±0.000005a	0.000068±0.000002a	0.00007±0.000003a	0.000055±0.00005a
5	2-keto-isovaleric acid	9.15794,0	141	759-05-7	0.000521±0.000007a	0.000513±0.000034a	0.000508±0.000013a	0.000546±0.000033b	0.000272±0.000242b
6	2-Methylglutaric Acid	14.5427,0	172	617-62-9	0.000141±0.000005b	0.000141±0.000005b	0.000136±0.000009b	0.000204±0.000009c	0±0c
7	3-hydroxybenzoic acid	16.9787,0	303	1999/6/9	0.000011±0.000002b	0.000014±0.000001a	0.000011±0b	0.000008±0.000001b	0±0b

8	3-hydroxybutyric acid	9.45,0	131	565-70-8	0.000006±0.000006c	0.00005±0.000001a	0.000062±0.000002a	0±0b	0.000033±0.000015b
9	3-Hydroxypropionic acid	9.80194,0	177	503-66-2	0.000308±0.000005b	0.000352±0.000007b	0.000497±0.000029a	0.000256±0.000006b	0.000291±0.000134b
10	3-Hexenedioic acid	15.9912,0	288	4436-74-2	0.000111±0c	0.000333±0.000008b	0.000518±0.000039a	0.000147±0.000006c	0.000135±0.000121c
11	4-hydroxybutyrate	11.4817,0	233	502-85-2	0.000122±0.000002a	0.000076±0.000002a	0.000095±0.000003a	0.000056±0.000004a	0.000116±0.000079a
12	4-Hydroxybenzoic acid	17.9032,0	267	99-96-7	0.000051±0.000003b	0.000094±0.000004a	0.000075±0.000002ab	0.000095±0.000006b	0.000044±0.00004b
13	4-Hydroxymandelic acid	19.8003,0	267	1198-84-1	0.000232±0.000002c	0.000304±0.000003b	0.00022±0.000015c	0.000388±0.000012d	0±0d
14	Aconitic Acid	19.5936,0	375	4023-65-8	0.000124±0.000002a	0.000129±0.000007a	0.000132±0.000011a	0.00016±0.000016a	0.000133±0.000091a
15	alpha-ketoglutaric acid	17.0982,0	198	328-50-7	0.000317±0.000019d	0.001147±0.000022b	0.000854±0.000058bc	0.001581±0.000033cd	0.00053±0.000474cd
16	Arachidic acid	27.8413,0	369	506-30-9	0.000039±0.000004a	0.000024±0.000003a	0.000019±0.000017a	0.000037±0.000005a	0.000029±0.000051a

17	Aspartic acid	16.11,0	174	56-84-8	0.000009±0.000008b	0.000022±0.000004a	0.000025±0a	0.000008±0.000013b	0±0b
18	Caffeic acid	24.5076,0	396	331-39-5	0.000441±0.000012ab	0.000473±0.000012ab	0.000516±0.000033a	0.000382±0.00001b	0.000275±0.000245b
19	Citramalic acid	15.4462,0	247	2306-22-1	0.000661±0.000019ab	0.000895±0.000032ab	0.001119±0.00008a	0.001045±0.000044b	0.000593±0.00053b
20	Citric acid	20.5935,0	363	5949-29-1	0.079295±0.000471a	0.079443±0.000928a	0.076751±0.004042a	0.08728±0.003587a	0.064445±0.057092a
21	D-Glyceric acid	12.8886,0	175	6000-40-4	0.001061±0.000929a	0.000419±0.000726a	0.000498±0.000862a	0.000354±0.000614a	0±0a
22	Ferulic acid	24.0038,0	338	1135-24-6	0.000243±0.000009a	0.000243±0.000004a	0.000253±0.00001a	0.000273±0.000011a	0.000201±0.000177a
23	Fumaric acid	13.5006,0	245	110-17-8	0.000143±0.000005ab	0.000164±0.000009ab	0.000112±0.000006ab	0.000179±0.00001b	0.000097±0.000086b
24	Galactonic acid	22.8179,0	275	576-36-3	0.000019±0.000003a	0.000017±0.000015a	0.00003±0.000004a	0.000013±0.00001a	0.000029±0.000024a
25	Gallic acid	22.3186,0	458	149-91-7	0.000412±0.000017a	0.000386±0.000002a	0.000334±0.000015b	0.000349±0.000027c	0.000204±0.000025c

26	Glucosaminic acid	23.2132,0	319	3646-68-2	0.000048±0.000002b	0.000055±0.000003b	0.000058±0.000001b	0.000058±0.000009a	0.006885±0.006071a
27	Glutaric Acid	14.4171,0	261	110-94-1	0.000023±0.000001b	0.000036±0.000001ab	0.000046±0.000005a	0.000026±0.000001ab	0.000026±0.000023ab
28	Gluconic acid	22.8086,0	333	526-95-4	0.008979±0.000224a	0.010935±0.000223a	0.012533±0.000475a	0.009266±0.000296a	0.019104±0.016289a
29	Glutamic acid	15.4462,0	247	2306-22-1	0.000661±0.000019ab	0.000895±0.000032ab	0.001119±0.00008a	0.001045±0.000044b	0.000593±0.00053b
30	Heptadecanoic acid	24.6171,0	100	506-12-7	0.000017±0.000015b	0.000027±0.000001ab	0.000035±0.000002a	0.00003±0c	0±0c
31	Itaconic acid	13.3432,0	215	97-65-4	0.000007±0.000001b	0.000019±0.000007ab	0.000017±0.000001a	0.000028±0.00002ab	0.000009±0.000008ab
32	Lactic acid	8.22389,0	117	50-21-5	0.010402±0.000093a	0.009427±0.000147ab	0.010741±0.000401a	0.008556±0.000265c	0.006768±0.002029c
33	Malic acid	15.7338,0	233	97-67-6	0.009715±0.000113ab	0.009207±0.000362b	0.00778±0.000551bc	0.012888±0.000317c	0.004798±0.004256c
34	Mucic acid	23.3232,0	292	526-99-8	0.000251±0.000003a	0.000295±0.000003a	0.000278±0.000009a	0.000273±0.000006a	0.000281±0.000248a

35	N-Acetyl-L-aspartic acid	18.3837,0	255	997-55-7	0.000013±0.000001b	0.000014±0.000001ab	0.000014±0.000001ab	0.000017±0.000002c	0±0c
36	N-Acetyl-L-phenyl alanine	20.1515,0	294	2018-61-3	0.000065±0.000004ab	0.000055±0.000002b	0.000057±0.000002b	0.000072±0.000005c	0.000012±0.000015c
37	Ornithine	20.432,0	435	70-26-8	0.000012±0.000001b	0.000016±0.000001a	0.000016±0.000001a	0.000013±0.000002c	0±0c
38	Oxalic acid	9.52,0	218	144-62-7	0.000156±0.000003a	0.000169±0.000015a	0.000193±0.000012a	0.000199±0.000029a	0.000177±0.00005a
39	Oxamic acid	9.52,0	218	144-62-7	0.000156±0.000003a	0.000169±0.000015a	0.000193±0.000012a	0.000199±0.000029a	0.000177±0.00005a
40	Palmitic acid	23.4697,0	313	1957/10/3	0.00663±0.000454b	0.005311±0.000359b	0.006394±0.000123b	0.006962±0.00007a	0.009988±0.00388a
41	Pelargonic acid	13.6921,0	174	112-05-0	0.00024±0.000001a	0.000255±0.000007a	0.000257±0.000004a	0.000278±0.000022b	0.000126±0.000112b
42	Pyruvic acid	8.0425,0	174	127-17-3	0.0013±0.000118a	0.001271±0.000016a	0.001007±0.000036a	0.000882±0.000061a	0.001507±0.001099a
43	Quinic acid	21.0442,0	346	77-95-2	0.034298±0.000369a	0.036766±0.000769a	0.033903±0.001613a	0.038808±0.001614b	0.010662±0.005905b

44	Saccharic acid	22.928,0	293	576-42-1	0±0b	0±0b	0±0b	0±0a	0.000075±0.000072a
45	Salicylic acid	16.0782,0	267	69-72-7	0.000019±0.000001a	0.000019±0.000001a	0.000023±0.000002a	0.000021±0.000001a	0.000017±0.000015a
46	Shikimic acid	20.1677,0	369	138-59-0	0.000004±0.000001a	0.000003±0a	0.000003±0.000001a	0.000003±0.000001b	0±0b
47	Sinapinic acid	25.7111,0	368		0±0b	0±0b	0.000019±0.000001a	0.000008±0.000015ab	0.000017±0.000015ab
48	Stearic acid	25.7378,0	341	1957/11/4	0.003869±0.000212a	0.00301±0.000075a	0.003672±0.0001a	0.003981±0.000079a	0.005319±0.002655a
49	Succinic acid	12.9058,0	147	110-15-6	0.071335±0.001057a	0.071024±0.001903a	0.065645±0.002487a	0.06528±0.026176a	0.038399±0.032939a
50	Tartaric acid	17.8561,0	349	133-37-9	0.000021±0.000001ab	0.00002±0.000001b	0.000021±0.000001ab	0.000023±0.000002a	0.000024±0.000003a
51	beta-Glutamic acid	17.5812,0	232	1948-48-7	0.000403±0.000032a	0.000367±0.000011a	0.000269±0.000022b	0.000229±0.00002c	0.000036±0.000036c
	**Alcohols**								
1	2-Butyne-1,4-diol	11.4644,0	159	110-65-6	0.000072±0.0000	0.000085±0.0000	0.000135±0.0000	0.00006±0.00000	0.00006±0.00005

					02b	03b	05a	3b	4b
2	2-Deoxyerythritol	12.4286,0	117	3068-00-6	0.000765±0.0000 14d	0.001571±0.0000 47b	0.001881±0.0000 87a	0.001392±0.0000 29c	0.000785±0.0001 76d
3	4-Methylbenzyl alcohol	11.8609,0	130	589-18-4	0.000183±0.0000 16a	0.000161±0.0000 33ab	0.000126±0.0000 08b	0.000135±0.0000 14b	0.000014±0.0000 12c
4	D-Arabitol	19.1494,0	307	488-82-4	0.005451±0.0001 66a	0.003505±0.0000 28b	0.003653±0.0002 55b	0.001335±0.0000 66c	0.001303±0.0019 25c
5	Dodecanol	16.8369,0	71	112-53-8	0.000146±0.0000 05b	0.000133±0.0000 14b	0.000151±0.0000 04b	0.000173±0.0000 13ab	0.000221±0.0000 78a
6	Ethanolamine	12.005,0	174	141-43-5	0.000109±0.0000 03ab	0.00009±0.00000 2b	0.000129±0.0000 09ab	0.000241±0.0000 05a	0.000189±0.0001 66ab
7	Mannitol	21.9317,0	319	87-78-5	0.00589±0.00008 b	0.007653±0.0001 89b	0.007198±0.0003 56b	0.007868±0.0002 49b	0.014382±0.0064 46a
8	Maltitol	31.4258,0	297	585-88-6	0.000019±0.0000 34a	0.000037±0.0000 32a	0±0a	0±0a	0.00002±0.00003 4a
9	myo-inositol	23.9133,0	305	87-89-8	0.037779±0.0002 17b	0.035089±0.0006 68b	0.0354±0.001536 b	0.0401±0.001685 ab	0.047649±0.0111 16a
10	Octadecanol	24.7591,0	327	112-92-5	0.000017±0.0000	0.000018±0.0000	0.000018±0.0000	0.000016±0.0000	0.000033±0.0000

					02a	05a	06a	03a	18a
11	Panthenol	21.2368,0	262	16485-10-2	0.008423±0.000281c	0.01113±0.000169b	0.013347±0.000418a	0.010637±0.00037b	0±0d
12	Phytol	24.8678,0	85	150-86-7	0.000225±0.000026b	0.00017±0.000007b	0.000222±0.000022b	0.000241±0.000004b	0.000392±0.000157a
13	Ribitol	19.1425,0	241	488-81-3	0.000002±0.000004a	0.000004±0.000004a	0.000003±0.000004a	0.000007±0.000002a	0.000003±0.000002a
14	Sorbitol	21.9669,0	289	50-70-4	0.00003±0.000004a	0.000028±0.000025a	0.000032±0.000028a	0.00003±0.000002a	0.000013±0.000011a
15	Threitol	15.8825,0	217	7493-90-5	0.000429±0.000004a	0.000273±0.000006c	0.000342±0.000021b	0.000247±0.000009c	0.000119±0.000059d
	**Aldehydes**								
1	4-hydroxy-3-methoxycinnamaldehyde	22.4406,0	175	458-36-6	0.000243±0.000002b	0.000213±0.000003b	0.000263±0.00001b	0.000207±0.000007b	0.000397±0.000111a
2	Butyraldehyde	11.9638,0	140	123-72-8	0.000005±0a	0.000005±0a	0.000005±0a	0.000006±0a	0.000003±0.000002b
3	Succinate semialdehyde	10.6297,0	144	692-29-5	0.000009±0ab	0.000003±0.000006b	0.00001±0a	0.00001±0.000001a	0.000004±0.000004b

	**Carbohydrates**								
1	1-Kestose	36.5436,0	437	470-69-9	0.000053±0.000004b	0.000056±0.000007b	0.000046±0.000003b	0.000036±0.000002b	0.000444±0.00026a
2	2-Deoxy-D-galactose	20.1827,0	464	1949-89-9	0.000142±0.000007c	0.000176±0.00001b	0.000212±0.000006a	0.000173±0.000009b	0±0d
3	2-deoxy-D-glucose	20.0143,0	307	154-17-6	0.000033±0.000009ab	0.000031±0.000002ab	0.000027±0.000003b	0.000038±0.000004a	0±0c
4	6-deoxy-D-glucose	19.0978,0	160	488-79-9	0.000382±0.000003b	0.000471±0.00001a	0.000423±0.000026ab	0.000442±0.000013a	0.000272±0.00005c
5	Altrose	21.7182,0	289	1990-29-0	0.000061±0.000053ab	0.000142±0.000002a	0.000105±0.000091a	0.00006±0.000052ab	0±0b
6	Cellobiose	30.2444,0	480	528-50-7	0.000244±0.00001b	0.000283±0.000003b	0.000193±0.000139b	0.000244±0.000009b	0.001711±0.00056a
7	Cellobiotol	30.7963,0	437	535-94-4	0.000158±0.000006b	0.00016±0.000007b	0.000099±0.000088b	0.000122±0.000023b	0.00089±0.000326a
8	Conduritol b epoxide	22.2136,0	332	6090-95-5	0.000503±0.00001c	0.000562±0.000017bc	0.000755±0.000031a	0.000612±0.000027bc	0.000672±0.000133ab
9	d-Glucoheptose	24.4367,0	103	62475-58-	0.00028±0.00000	0.000324±0.0000	0.000367±0.0000	0.000439±0.0000	0.000456±0.0000

				5	4b	16b	22ab	3a	96a
10	Digitoxose	17.5533,0	277	527-52-6	0.000258±0.000005a	0.000217±0.000008b	0.000231±0.000018b	0.000192±0.000006c	0±0d
11	Fructose-6-phosphate	26.3623,0	262	643-13-0	0.000618±0.000007c	0.000751±0.000039a	0.000778±0.000039a	0.000672±0.000013b	0±0d
12	Fructose 2,6-biphosphate	25.2962,0	342	79082-92-1	0.000085±0.000002bc	0.000106±0.000006ab	0.000129±0.000004a	0.000069±0.000005c	0.000039±0.000035d
13	Galactinol	32.25,0	204	3687-64-7	0.000368±0.000004c	0.00103±0.000011a	0.001162±0.00008a	0.000827±0.000016b	0.000794±0.000143b
14	Gentiobiose	31.3353,0	361	554-91-6	0.002943±0.000042c	0.003389±0.000114abc	0.003543±0.000258ab	0.003746±0.000028a	0.0031±0.000473bc
15	Glucose-1-phosphate	19.8374,0	226	59-56-3	0.002944±0.000063a	0.002767±0.000384a	0.002653±0.000087a	0.003013±0.000332a	0.001224±0.001086b
16	Isomaltose	32.0717,0	361	499-40-1	0.000658±0.000014ab	0.000573±0.000008b	0.000622±0.000041ab	0.000619±0.000014ab	0.000713±0.000099a
17	Lactose	30.3015,0	406	63-42-3	0.000041±0.000001a	0.000044±0a	0.000051±0.000002a	0.000041±0.000001a	0.000043±0.000038a
18	Lactulose	30.184,0	107	4618-18-2	0.000043±0.0000	0.000023±0.0000	0.000022±0.0000	0.000032±0a	0.000024±0.0000

					03a	18a	19a		22a
19	Loganin	32.2178,0	236	18524-94-2	0.000125±0.000003a	0.000105±0.000002b	0.000106±0.000008b	0.000117±0.000004ab	0.000065±0.000014c
20	Lyxose	18.2232,0	307	1114-34-7	0.001161±0.000021b	0.001067±0.00002b	0.001059±0.00007b	0.001224±0.000051b	0.001557±0.000385a
21	Maltose	30.5278,0	361	69-79-4	0.011497±0.000262c	0.014752±0.000188b	0.015918±0.001779b	0.015878±0.000521b	0.019196±0.003147a
22	Maltotriitol	40.33,0	479	32860-62-1	0.000028±0.000004a	0.000031±0.000002a	0.000031±0.000003a	0.000027±0.000007a	0.000007±0.000007b
23	Maltotriose	38.7517,0	319	1109-28-0	0.000034±0.000001a	0.000024±0.000005a	0.000025±0.000017a	0.000022±0.000005a	0.000017±0.000006a
24	Melibiose	31.7897,0	361	66009-10-7	0.003807±0.00006ab	0.00332±0.000043b	0.003762±0.000196ab	0.003533±0.000113ab	0.00458±0.001324a
25	Melezitose	37.6925,0	437	597-12-6	0.000041±0.000002b	0.000043±0.000002b	0.000048±0.000001b	0.000034±0.000002b	0.000066±0.000021a
26	Palatinose	31.3983,0	307	15132-06-6	0.000517±0.000006b	0.000627±0.000008a	0.000693±0.000038a	0.000472±0.000011b	0.00065±0.000099a
27	Palatinitol	32.3021,0	308	64519-82-	0.000051±0.0000	0.000047±0.0000	0.000045±0.0000	0.000052±0.0000	0.000072±0.0000

				0	02a	04a	04a	03a	65a
28	Raffinose	36.4039,0	361	512-69-6	0.003429±0.000077b	0.00276±0.000138b	0.00272±0.000182b	0.002505±0.000081b	0.010281±0.00477a
29	Ribose	18.3261,0	307	24259-59-4	0.010538±0.00021b	0.012063±0.000205a	0.011612±0.000851ab	0.012974±0.000557a	0.007535±0.00127c
30	Ribose-5-phosphate	24.0854,0	315	4151-19-3	0.000028±0.000002a	0.000015±0.000013a	0.000042±0.000003a	0.000022±0.000019a	0.000043±0.000039a
31	Ribulose-5-phosphate	23.9781,0	362	4151-19-3	0±0c	0.00009±0.000003a	0.000021±0.000037bc	0.000072±0.000062ab	0±0c
32	Salicin	28.3017,0	321	138-52-3	0.00037±0.000006a	0.000375±0.000006a	0.000388±0.000023a	0.000408±0.000012a	0.000356±0.000144a
33	Sophorose	30.9364,0	319	534-46-3	0.001098±0.000022d	0.001966±0.000029b	0.001895±0.000148b	0.002368±0.000074a	0.001561±0.000277c
34	Sucrose	29.6381,0	361	57-50-1	0.006099±0.000031b	0.003603±0.00002b	0.004165±0.000406b	0.005328±0.000205b	0.013508±0.003215a
35	Sucrose-6-Phosphate	32.9777,0	364	36064-19-4	0.000003±0.000004a	0.000004±0.000004a	0.000012±0a	0.000012±0.000003a	0.000006±0.00001a
36	Tagatose	20.886,0	307	87-81-0	0.0001±0.000004	0.00006±0.00005	0.000113±0.0000	0.000073±0.0000	0.023924±0.0414

					a	2a	09a	04a	37a
37	Talose	21.7457,0	319	2595-98-4	0.006894±0.000082a	0.004616±0.003998a	0.007079±0.000373a	0.006759±0.000255a	0.023151±0.040098a
38	Trehalose	30.6467,0	362	99-20-7	0.005347±0.000158d	0.011962±0.000123b	0.011059±0.00078bc	0.01408±0.00036a	0.009947±0.001449c
39	Trehalose-6-phosphate	34.7339,0	368	4484-88-2	0.00006±0.000001a	0.00006±0.000001a	0.000066±0.000003a	0.000068±0.000007a	0.000061±0.000052a
40	Xylose	18.2392,0	276	6763-34-4	0.000088±0.000002b	0.000079±0.000004c	0.000075±0.000004c	0.000111±0.000003a	0.000006±0.000003d
41	alpha-D-glucosamine 1-phosphate	20.2614,0	113	2152-75-2	0.000069±0.000004a	0.000021±0.000036a	0.000082±0.000005a	0.00008±0.000004a	0.000039±0.000068a
	**Others**								
1	1,2-Cyclohexanedione	18.4753,0	261	765-87-7	0.000027±0.000001a	0.00002±0a	0.000031±0.000002a	0.000015±0.000002a	0.000013±0.000022a
2	1,4-Cyclohexanedione	12.8281,0	141	637-88-7	0.000014±0.000001a	0.000012±0.000001a	0.000014±0.000001a	0.000016±0.000001a	0.000016±0.000008a
3	2-hydroxypyridine	7.81029,0	152	142-08-5	0.002527±0.000079a	0.002479±0.000126a	0.002622±0.000027a	0.002829±0.000213a	0.003276±0.002915a

4	2,3-Dihydroxypyridine	12.8594,0	240	16867-04-2	0.000036±0.000003a	0.000032±0.000001a	0.000032±0.000002a	0.000038±0.000003a	0.000078±0.00007a
5	2,4-diaminobutyric acid	15.2097,0	51	305-62-4	0.00007±0.000003a	0.000066±0.000008a	0.00006±0.000004ab	0.000068±0.00001a	0.000044±0.000019b
6	2-mercaptoethanesulfonic acid	17.6279,0	337	3375-50-6	0.000017±0.000003a	0.000006±0.000006b	0.00001±0.000001ab	0.000007±0.000007b	0.000003±0.000006b
7	3-Aminoisobutyric acid	10.9563,0	102	144-90-1	0.000294±0.000027a	0.000183±0.000062b	0.000143±0.000017b	0.000185±0.000037b	0±0c
8	3-Cyanoalanine	13.9583,0	201	6232-19-5	0.000009±0.000001a	0.000009±0.000001a	0.000009±0.000001a	0.000009±0.000001a	0±0b
9	3-Hydroxynorvaline	15.0656,0	233	2280-42-4	0.000313±0.000006b	0.000234±0.00001c	0.00022±0.000014c	0.000348±0.000007a	0.000026±0.000023d
10	3-Hydroxypyridine	9.68765,0	152	109-00-2	0.000286±0.000011b	0.000263±0.00001b	0.000284±0.000008b	0.000305±0.000028b	0.001502±0.001326a
11	3-Methylamino-1,2-propanediol	10.9271,0	187	40137-22-2	0.000034±0.000001a	0.000042±0a	0.000048±0.000003a	0.000034±0.000003a	0.00003±0.000026a
12	3-Methyloxindole	18.969,0	364	1504-06-9	0.000014±0.000001ab	0.000014±0ab	0.000015±0.000002a	0.000013±0b	0±0c

13	3-hydroxy-3-methylglutaric acid	17.4785,0	363	503-49-1	0.00006±0a	0.00007±0.000005a	0.000071±0.000006a	0.000065±0.000003a	0.000039±0.000035a
14	3-hydroxy-L-proline	15.3074,0	389		0.000017±0.000001a	0.000017±0.000001a	0.000018±0.000001a	0.000022±0.000002a	0.000017±0.000015a
15	3-Phenyllactic acid	17.2115,0	193	828-01-3	0.00042±0.000009a	0.00022±0.000004bc	0.000179±0.000014cd	0.000283±0.000013b	0.000117±0.000104d
16	4-Hydroxy-6-methyl-2-pyrone	15.8158,0	71	675-10-5	0.00036±0.000009b	0.000323±0.000013b	0.000376±0.000012ab	0.000412±0.00001ab	0.000528±0.000183a
17	4-Hydroxycyclohexanecarboxylic acid	15.7912,0	275	3685-22-1	0.000033±0.000002a	0.000035±0.000001a	0.000035±0.000002a	0.000044±0.000002a	0.000017±0.000016b
18	4-Hydroxyquinazoline	16.6819,0	217	491-36-1	0.000197±0.000012a	0.000233±0.000009a	0.00025±0.00002a	0.000288±0.000014a	0.000344±0.000245a
19	4-hydroxypyridine	10.2268,0	152	626-64-2	0.000087±0.000003a	0.000123±0.000002a	0.000127±0.000005a	0.000131±0.000007a	0.000085±0.000075a
20	5-Aminovaleric acid	18.4075,0	220	660-88-8	0.000002±0.000004a	0.000004±0.000004a	0.000006±0.000005a	0.000003±0.000005a	0±0a
21	5-Dihydrocortisol	34.4789,0	105	516-41-6	0.000024±0.000007a	0.000026±0.000008a	0.000029±0.000004a	0.000038±0.000001a	0.00003±0.000017a

22	5-Hydroxyindole-3-acetic acid	25.4076,0	422	54-16-0	0.000012±0.000001ab	0.000011±0b	0.000017±0.000002a	0.000015±0.000003ab	0.000005±0.000006c
23	5-alpha-Cholestan-3-one	35.5218,0	236	566-88-1	0.000003±0.000005a	0.000007±0.000001a	0.000008±0a	0.000005±0.000005a	0.000008±0.000007a
24	21-hydroxypregnenolone	33.515,0	361	1164-98-3	0±0a	0.000001±0.000002a	0±0a	0.000002±0.000003a	0.000003±0.000003a
25	Acetol	18.985,0	85	116-09-6	0.000177±0.000008b	0.000145±0.00001b	0.000193±0.000023b	0.000216±0.00001b	0.000356±0.000126a
26	alpha-Aminoadipic acid	18.9067,0	288	542-32-5	0.000081±0.000007b	0.000082±0.000003b	0.000085±0.000013b	0.000107±0.000007a	0±0c
27	Aminomalonic acid	15.4168,0	145	1068-84-4	0.001242±0.000024a	0.000419±0.000013bc	0.000394±0.00002c	0.000531±0.000021b	0.000149±0.000133d
28	Arbutin	28.9065,0	361	497-76-7	0.00026±0.000002a	0.000139±0.000121a	0.000207±0.000018a	0.000262±0.000007a	0.000321±0.000189a
29	Atrazine-2-hydroxy	21.8497,0	356	2163-68-0	0.000496±0.000017b	0.000436±0.000022c	0.000626±0.00005a	0.000583±0.000024a	0±0d
30	Benzamide	15.0494,0	174	55-21-0	0.000034±0.000001a	0.000033±0.000002a	0.000031±0.000003a	0.000038±0.000001a	0.000011±0.00001b

31	beta-Alanine	14.8062,0	174	107-95-9	0.000655±0.000016a	0.000665±0.000036a	0.000654±0.000025a	0.000732±0.000049a	0.000324±0.000286b
32	beta-Mannosylglycerate	25.2762,0	204	164324-35-0	0.000209±0.000013a	0.000189±0.000009a	0.000204±0.000013a	0.000195±0.000005a	0.000175±0.000153a
33	Biuret	19.8565,0	200	108-19-0	0.000008±0.000007a	0.000012±0.000001a	0.000012±0.000002a	0.000014±0.000002a	0±0b
34	Biphenyl	14.6629,0	232	92-52-4	0.000002±0.000002a	0±0b	0±0b	0.000001±0.000001ab	0±0ab
35	Canavanine	11.2712,0	230	543-38-4	0.000064±0.000006a	0.000083±0.000001a	0.000089±0.000005a	0.000091±0.000003a	0.00006±0.000053a
36	Carnitine	11.2221,0	203	541-15-1	0.000036±0.000001a	0.000037±0.000001a	0.000046±0.000003a	0.000034±0.000001a	0.000038±0.000033a
37	Chlorogenic Acid	33.8765,0	345	327-97-9	0.000331±0.000003a	0.00054±0.000014a	0.000577±0.000035a	0.000359±0.000025a	0.000591±0.000526a
38	Cholestan-3beta-ol	34.8953,0	336	80-97-7	0.000015±0.000001a	0.000014±0.000001a	0.000012±0.000001a	0.000016±0.000001a	0.000014±0.000012a
39	Citrulline	19.6669,0	85	372-75-8	0.000042±0.000005a	0.000035±0.000003ab	0.000046±0.000005a	0.000047±0.000004a	0.000015±0.000027b

40	Cyclohexylsulfamic acid	18.6491,0	294	100-88-9	0.000031±0.000002a	0.000028±0.000004a	0.000033±0.000001a	0.000034±0.000002a	0.000044±0.000038a
41	Cycloleucine	10.835,0	57	52-52-8	0.00005±0.000003a	0.000047±0.000001a	0.000052±0.000001a	0.000058±0.000002a	0.000087±0.000054a
42	D-(glycerol 1-phosphate)	19.735,0	292	1957/3/4	0.000437±0.00001a	0.000476±0.000009a	0.00057±0.000014a	0.000456±0.000023a	0.000413±0.00037a
43	D-erythro-sphingosine	27.7585,0	361	123-78-4	0.000062±0.000005a	0.000066±0.000003a	0.000074±0.000004a	0.000073±0.000004a	0.000063±0.000056a
44	D-galacturonic acid	22.3539,0	333	685-73-4	0.010789±0.000074b	0.017973±0.000326a	0.017682±0.001004a	0.011275±0.000466b	0.009219±0.007163b
45	Dihydroxyacetone	11.7518,0	174	62147-49-3	0.000127±0.000001ab	0.000129±0.000004ab	0.000099±0.000007b	0.000157±0.000008a	0.000047±0.000041c
46	DL-Anabasine	14.3597,0	254		0.000011±0.000009b	0.000026±0a	0.000027±0.000001a	0.00003±0.000003a	0.000004±0.000007b
47	DL-dihydrosphingosine	28.3697,0	204	13552-09-5	0.000128±0.000003b	0.000741±0.001045b	0.00015±0.000014b	0.002119±0.000038a	0.000729±0.001006b
48	dl-p-Hydroxyphenyllactic acid	21.6279,0	312	6482-98-0	0.000229±0.000199a	0.000341±0.000008a	0.000324±0.000018a	0.000362±0.000018a	0±0b

49	Epigallocatechin	32.33,0	239	970-74-1	0.000021±0.000002a	0.000018±0.000001a	0.000019±0.000001a	0.00002±0.000001a	0.000017±0.000015a
50	Erythrose	15.1481,0	350	583-50-6	0.000063±0.000004a	0.000064±0.000014a	0.000059±0.000005a	0.000062±0.000003a	0.000012±0.000003b
51	Glutaconic acid	19.4936,0	359	1724-02-3	0.001659±0.000175b	0.001747±0.000054b	0.002035±0.000119ab	0.002295±0.000217a	0.000808±0.000441c
52	Glutamine	16.5275,0	71	56-85-9	0.000602±0.00001b	0.000533±0.000022b	0.000622±0.000037b	0.00069±0.000018ab	0.000953±0.000347a
53	Guanidinosuccinic acid	18.5839,0	57	6133-30-8	0.000197±0.000024a	0.000103±0.000095a	0.000112±0.000097a	0.000197±0.000016a	0.000083±0.000143a
54	Hydroxyurea	11.5056,0	248	127-07-1	0.000026±0.000002a	0.000025±0a	0.000027±0.000001a	0.000028±0.000001a	0.000016±0.000015a
55	Iminodiacetic acid	16.3925,0	174	142-73-4	0.000041±0.000001b	0.000018±0.000015c	0.000049±0.000003b	0.000083±0.000004a	0.000018±0.000016c
56	Inosine	29.0222,0	349	58-63-9	0.000022±0a	0.000026±0.000002a	0.000025±0.000003a	0.00002±0.000001a	0.000021±0.000009a
57	Isopropyl-beta-D-thiogalactopyranosid	23.6594,0	217	367-93-1	0.000429±0.000006ab	0.000414±0.000005b	0.000393±0.000023b	0.000475±0.000013a	0.000147±0.000056c

	e								
58	Kyotorphin	28.9693,0	297	70904-56-2	0.000021±0.000001a	0.000021±0.000003a	0.000023±0.000001a	0.000021±0.000001a	0±0b
59	Levoglucosan	18.8139,0	231	498-07-7	0.000109±0.000003b	0.000117±0.000003b	0.000113±0.000009b	0.000127±0.000005b	0.000245±0.000045a
60	Lipoic acid	22.3889,0	71	1077-28-7	0.000379±0.000023a	0.000355±0.000014a	0.000283±0.000202a	0.000449±0.000007a	0.000498±0.000206a
61	Maleamate	16.7165,0	198	557-24-4	0.000016±0.000001d	0.000046±0b	0.000035±0.000004bc	0.000063±0.000001a	0.000021±0.000019cd
62	Maleimide	8.89618,0	244	541-59-3	0.000012±0.000001a	0.000011±0.000001a	0.000015±0.000001a	0.000017±0.000001a	0.000018±0.000016a
63	Malonamide	14.8879,0	89	108-13-4	0.000016±0.000002a	0.000011±0.000009ab	0.000017±0.000001a	0.000006±0.00001ab	0±0b
64	N-Acetyl-5-hydroxytryptamine	28.4562,0	292	1210-83-9	0.000041±0.000003a	0.000039±0a	0.000038±0.000004a	0.00005±0.000003a	0.000035±0.000031a
65	N-Acetyl-beta-D-mannosamine	24.0972,0	319	7772-94-3	0.001407±0.000009a	0.001471±0.000052a	0.00153±0.000067a	0.00156±0.000053a	0.001448±0.00032a
66	N-Acetyl-D-galacto	23.7589,0	202	14215-68-	0.000011±0c	0.000013±0.0000	0.000017±0.0000	0.000026±0.0000	0.00006±0.00001

	samine			0		01c	02bc	02b	4a
67	N-Ethylglycine	14.4853,0	174	627-01-0	0.00092±0.000008a	0.000926±0.000034a	0.000897±0.000041a	0.001023±0.000169a	0.000285±0.000254b
68	N-Methyl-DL-alanine	10.3391,0	202	600-21-5	0.000007±0a	0.000004±0.000004a	0.000008±0a	0.000007±0.000001a	0.000007±0.000007a
69	N-(2-hydroxyethyl)-iminodiacetic acid	20.6163,0	249	93-62-9	0.000223±0.000003c	0.000243±0.000004b	0±0d	0.000256±0.00001a	0±0d
70	Naphthalene	11.1438,0	57	91-20-3	0.000192±0.000026a	0.000197±0.000006a	0.000219±0.000008a	0.000241±0.000012a	0.000156±0.000143a
71	O-methylthreonine	10.3788,0	322	2076-57-5	0.000048±0.000001a	0.000041±0.000004a	0.000043±0.000003a	0.000046±0.000002a	0.00001±0.00001b
72	Oxamide	13.0747,0	71	471-46-5	0.000969±0.000019ab	0.000867±0.000031b	0.000966±0.000043ab	0.00106±0.000041ab	0.001229±0.000393a
73	Oxoproline	16.2609,0	156	98-79-3	0.000412±0.00001a	0.000277±0.000025a	0.000436±0.000031a	0.000395±0.000011a	0.000359±0.000315a
74	Phosphate	12.0965,0	299	7664-38-2	0.032466±0.000381a	0.029949±0.000708a	0.029207±0.001571a	0.037165±0.000826a	0.02255±0.019882a
75	Piceatannol	28.6612,0	181	10083-24-	0.000008±0.0000	0.00001±0.00000	0.000014±0.0000	0.000024±0.0000	0.000025±0.0000

				6	14a	9a	12a	01a	25a
76	Purine riboside	26.9867,0	259		0.002677±0.00002a	0.002762±0.000118a	0.002437±0.000159a	0.002867±0.000029a	0.001533±0.000562b
77	Resorcinol	14.0376,0	240	108-46-3	0.000119±0.000002a	0.000113±0.000004a	0.00012±0.000004a	0.000131±0.000009a	0.000147±0.00013a
78	Sarcosine	9.63071,0	116	107-97-1	0.000033±0.000004a	0.000023±0.00002a	0.00003±0.000005a	0.000031±0.000007a	0±0b
79	Threo-beta-hyrdoxyaspartate	17.405,0	292	7298-99-9	0.000022±0.000001a	0.000021±0.000001a	0.000023±0a	0.000014±0.000012a	0.000024±0.000042a
80	Threonic acid	16.8115,0	292	7306-96-9	0.000397±0.000006ab	0.000426±0.00002ab	0.000469±0.000038a	0.000466±0.000019a	0.000246±0.00022b
81	Threose	15.2395,0	288	95-44-3	0.000002±0a	0±0b	0.000002±0.000001ab	0.000002±0.000002ab	0.000001±0.000001ab
82	Thymidine	17.6383,0	262	50-89-5	0.005101±0.000157c	0.004545±0.00012c	0.006582±0.000826b	0.006739±0.000248b	0.007789±0.000859a
83	trans-4-hydroxy-L-proline	16.3143,0	230	51-35-4	0.000035±0.000002a	0.000016±0.000002c	0.000013±0.000001d	0.000026±0.000002b	0±0e
84	Uracil	13.2524,0	238	66-22-8	0.000066±0.0000	0.000061±0.0000	0.000061±0.0000	0.000065±0.0000	0.000039±0.0000

					03a	06a	02a	01a	35a
85	Urea	11.4311,0	68	57-13-6	0.000306±0.000008a	0.00029±0.000014a	0.0003±0.00001a	0.000328±0.00002a	0.000376±0.000111a
86	Uridine	27.9969,0	450	58-96-8	0.000019±0.000001b	0.000017±0.000003b	0.000018±0.000002b	0.000026±0.000002a	0.000003±0.000002c
87	(+)-catechin	31.6869,0	368	154-23-4	0.000013±0.000001a	0.000011±0.000001a	0.000014±0.000001a	0.000008±0.000007a	0.000018±0.000016a
88	(+/-)-Taxifolin	32.5232,0	473	480-18-2	0.000015±0.000001a	0.000017±0.000002a	0.000022±0.000002a	0.00002±0.000003a	0.000006±0.000009b
89	4-Cholesten-3-one	35.0089,0	439	601-57-0	0.000012±0.000002a	0.000008±0.000001ab	0.000003±0.000006b	0.000011±0.000001a	0.000002±0.000004b
	**Esters**								
1	2-Monopalmitin	28.72,0	219	23470-00-0	0.000253±0.000022a	0.000264±0.000005a	0.000256±0.000019a	0.000268±0.000005a	0.000273±0.000076a
2	Carbobenzyloxy-L-leucine	11.6443,0	128	2018-66-8	0.000006±0.00001a	0.000005±0.000009a	0.000011±0.00001a	0.000005±0.000009a	0±0a
3	D-erythronolactone	14.76,0	257	15667-21-7	0.000037±0.000002a	0.000043±0.000002a	0.000053±0.000007a	0.00005±0.000002a	0.00005±0.000045a

4	Flavin adenine dinucleotide	19.3056,0	273	146-14-5	0.000049±0.000002ab	0.000054±0.000002a	0.000054±0.000004a	0.000055±0.000004a	0.000029±0.000025b
5	Gluconic lactone	21.5782,0	334	90-80-2	0.00694±0.000145a	0.007288±0.000065a	0.005924±0.005132a	0.008033±0.000362a	0±0b
6	Lactamide	10.1475,0	116	2043-43-8	0.000067±0.000006a	0.000033±0.000001b	0.00003±0.000002b	0.000028±0.000002b	0.000005±0.000009c
7	Lactobionic Acid	30.75,0	495	96-82-2	0.000004±0.000005ab	0.000009±0.000008ab	0.000008±0.000006ab	0.000012±0.000001a	0±0b
8	L-Gulonolactone	21.6873,0	465	1128-23-0	0.000071±0.000004a	0.000081±0.000002a	0.000075±0.000008a	0.000072±0.000006a	0±0b
9	Lyxonic acid, 1,4-lactone	19.4118,0	364	15384-34-6	0.000031±0.000003a	0.000034±0.000001a	0.000035±0.000003a	0.000038±0.000002a	0.000021±0.000019a
10	Methyl Phosphate	10.3892,0	241	812-00-0	0.000007±0a	0.000004±0.000004a	0.000004±0.000003a	0.000005±0.000005a	0.000005±0.000005a
11	Ribonic acid, gamma-lactone	18.6168,0	364	5336/8/3	0.000013±0.000001a	0.000011±0.000001a	0.000012±0.000002a	0.000012±0.000001a	0.000008±0.000007a
12	Uridine monophosphate	31.519,0	472	58-97-9	0±0a	0.000019±0.000001a	0.000016±0.000014a	0.000015±0.000013a	0.000008±0.000014a
